# Regulation of RUNX1 dosage is crucial for efficient blood formation from hemogenic endothelium

**DOI:** 10.1242/dev.149419

**Published:** 2018-03-01

**Authors:** Michael Lie-A-Ling, Elli Marinopoulou, Andrew J. Lilly, Mairi Challinor, Rahima Patel, Christophe Lancrin, Valerie Kouskoff, Georges Lacaud

**Affiliations:** 1 Stem Cell Biology Group, Cancer Research UK Manchester Institute, The University of Manchester, Wilmslow Road, Manchester M20 4BX, UK; 2 Stem Cell Hematopoiesis, Cancer Research UK Manchester Institute, The University of Manchester, Wilmslow Road, Manchester M20 4BX, UK; 3 EMBL Rome, Epigenetics and Neurobiology Unit, Campus Adriano Buzzati-Traverso Via Ramarini 32, 00015 Monterotondo, Italy

**Keywords:** RUNX1, Hemogenic endothelium, Dosage, EHT, CBFβ, SOX7

## Abstract

During ontogeny, hematopoietic stem and progenitor cells arise from hemogenic endothelium through an endothelial-to-hematopoietic transition that is strictly dependent on the transcription factor RUNX1. Although it is well established that RUNX1 is essential for the onset of hematopoiesis, little is known about the role of RUNX1 dosage specifically in hemogenic endothelium and during the endothelial-to-hematopoietic transition. Here, we used the mouse embryonic stem cell differentiation system to determine if and how RUNX1 dosage affects hemogenic endothelium differentiation. The use of inducible *Runx1* expression combined with alterations in the expression of the RUNX1 co-factor CBFβ allowed us to evaluate a wide range of RUNX1 levels. We demonstrate that low RUNX1 levels are sufficient and necessary to initiate an effective endothelial-to-hematopoietic transition. Subsequently, RUNX1 is also required to complete the endothelial-to-hematopoietic transition and to generate functional hematopoietic precursors. In contrast, elevated levels of RUNX1 are able to drive an accelerated endothelial-to-hematopoietic transition, but the resulting cells are unable to generate mature hematopoietic cells. Together, our results suggest that RUNX1 dosage plays a pivotal role in hemogenic endothelium maturation and the establishment of the hematopoietic system.

## Abstract (precis)

**Summary:** At the onset of hematopoiesis, regulation of RUNX1 dosage is crucial for the successful maturation of hemogenic endothelium and for both the initiation and completion of the endothelial-to-hematopoietic transition.

## INTRODUCTION

During development, the first definitive blood cells arise from a specialized endothelium (hemogenic endothelium or HE), via a process termed endothelial-to-hematopoietic transition (EHT). EHT has been elegantly visualized both *in vivo* and *in vitro* using multiple vertebrate model systems (Bertrand et al., 2010; Boisset et al., 2010; Eilken et al., 2009; Jaffredo et al., 1998; Kissa and Herbomel, 2010; Lam et al., 2010; Lancrin et al., 2009). The transcription factor RUNX1 is crucial for EHT and the emergence of definitive blood cells from HE (Chen et al., 2009; Kissa and Herbomel, 2010; Lacaud et al., 2002; Lancrin et al., 2009; North et al., 1999). Within the context of the definitive adult blood system, alterations in RUNX1 dosage or activity have been associated with several blood-related disorders with both reduction (thrombocytopenia, myelodysplastic syndrome) and gain (Down syndrome hematopoietic disorders) of functional *Runx1* alleles leading to abnormalities (Banno et al., 2016; De Vita et al., 2010; Rio-Machin et al., 2012; Song et al., 1999). RUNX1 dosage also plays a crucial role in the maintenance of leukemias harboring core-binding factor-related translocations (Ben-Ami et al., 2013; Goyama et al., 2013; Ptasinska et al., 2014; Yanagida et al., 2005). RUNX1 dosage has also been extensively studied in ontogeny, with several studies clearly establishing that *Runx1* haploinsufficiency or mutations result in a decrease in generation of hematopoietic stem and/or progenitor cells both *in vitro* and *in vivo* (Cai et al., 2000; Lacaud et al., 2002, 2004; Matheny et al., 2007; Wang et al., 1996a). However, little is known about the precise role of RUNX1 dosage in HE and during EHT at the onset of hematopoiesis.

*Runx1* transcription is controlled by two alternative *Runx1* promoters that generate transcripts coding for the two main RUNX1 isoforms (Miyoshi et al., 1995). The P1, or distal, promoter controls the expression of the distal RUNX1 isoform RUNX1C, and the P2, or proximal, promoter controls the proximal isoform RUNX1B. On a protein level the two isoforms are mostly identical and only differ in their N-terminal region (Fujita et al., 2001; Miyoshi et al., 1995). The dual promoter structure and the difference in N-terminal amino acid sequence are conserved across all RUNX genes and also across different mammalian species (Levanon and Groner, 2004). Although clear biochemical differences between the two isoforms remain relatively poorly defined (Bonifer et al., 2017; Nieke et al., 2017), specific expression patterns for each isoform in adult hematopoiesis and different requirements in megakaryocytic and lymphoid lineage commitment have been demonstrated (Brady et al., 2013; Challen and Goodell, 2010; Draper et al., 2017, 2016; Telfer and Rothenberg, 2001).

*Runx1* P2 promoter activity starts early during hematopoietic development and is detected in HE, in which it is the sole active *Runx1* promoter in mice (Bee et al., 2009; Sroczynska et al., 2009a) indicating that the RUNX1B isoform is responsible for the initiation of EHT. Experiments in mice have demonstrated that lowering the levels of RUNX1B by creating *Runx1* heterozygote knockouts or by attenuating P2 proximal promoter activity does not dramatically affect the onset of hematopoiesis as all these animals develop to term (Bee et al., 2010; North et al., 1999; Pozner et al., 2007; Wang et al., 1996a). However, there are some indications that the RUNX1 levels change as the cells differentiate from hemangioblasts (HBs) via HE to the first CD41 (ITGA2B)^+^ hematopoietic progenitors (HPs). One line of evidence was provided by Swiers et al. who analyzed single cells derived from +23*Runx1* enhancer-reporter transgenic mice (23GFP) (Swiers et al., 2013). In this study, *Runx1* mRNA expression was found to be lower in embryo-derived 23GFP^+^ HE cells compared with CD41^+^ HPs (Swiers et al., 2013). In contrast to P2, the *Runx1* P1 promoter is activated later in development during EHT in committed CD41^+^ HPs (Bee et al., 2009; Sroczynska et al., 2009a). In the adult hematopoietic system, P1 is the dominant active promoter (Bee et al., 2009; Draper et al., 2016).

Several transcription factors have been shown to regulate RUNX1 protein activity. CBFβ is a crucial RUNX1 co-factor that heterodimerizes with RUNX1, enhances its DNA-binding affinity and protects it from degradation (Huang et al., 2001; Nagata et al., 1999; Tang et al., 2000). *Cbfb* knockout is embryonic lethal and shows an almost identical phenotype to that of *Runx1* knockout embryos (Niki et al., 1997; Okuda et al., 1996; Sasaki et al., 1996; Wang et al., 1996a,b). RUNX1 protein is barely detectable in these *Cbfb*^−/−^ embryos, suggesting that this phenotype is mainly caused by a reduction in RUNX1 levels (Huang et al., 2001; Yokomizo et al., 2008). More recently, SOXF transcription factor family members SOX7 and SOX17 have emerged as novel important regulators of RUNX1 activity. These transcription factors have established roles in vasculogenesis and angiogenesis, suggesting that they are potentially able to interact with RUNX1 in the context of HE development (Lilly et al., 2016b). Indeed, both SOX7 and SOX17 are co-expressed with RUNX1 during a narrow temporal window of hematopoietic development that encompasses HE (Clarke et al., 2013; Costa et al., 2012; Gandillet et al., 2009; Lizama et al., 2015). Furthermore, both factors have been proposed to be negative regulators of RUNX1 and overexpression of either transcription factor can block hematopoietic development (Clarke et al., 2013; Costa et al., 2012; Gandillet et al., 2009; Lilly et al., 2016a; Lizama et al., 2015).

Here, we sought to define the role and importance of RUNX1B dosage in HE and EHT. To circumvent the limitations of *in vivo* gene dosage experiments, we took advantage of the *in vitro* mouse embryonic stem cell (mESC) differentiation system. Mouse ESCs have been shown to recapitulate key events of early embryonic yolk sac hematopoiesis, including HE formation and differentiation (Choi et al., 1998; Eilken et al., 2009; Frame et al., 2016; Lancrin et al., 2010; Sroczynska et al., 2009b). To evaluate the effects of different RUNX1B expression levels on HE and EHT, we utilized an mESC line in which *Runx1b* transcription is under control of a doxycycline inducible tet-on system (Lancrin et al., 2009). Analyses of this line in the presence of either a wild-type or a disrupted *Cbfb* locus allowed us to evaluate the effects of a wide range of RUNX1B levels on blood cell development. Overall, our results indicate that RUNX1B is required for both the initiation and the completion of the EHT. Initially low levels of RUNX1B in HE are crucial for the maturation of the HE and the initiation of EHT. Circumventing HE maturation by increasing RUNX1 levels results in abortive differentiation, highlighting the need for a phase of low level of RUNX1 expression in the HE to initiate EHT successfully.

## RESULTS

### *Runx1b* transcripts are low and *Runx1c* transcripts are absent prior to EHT

**Fig. 1.**
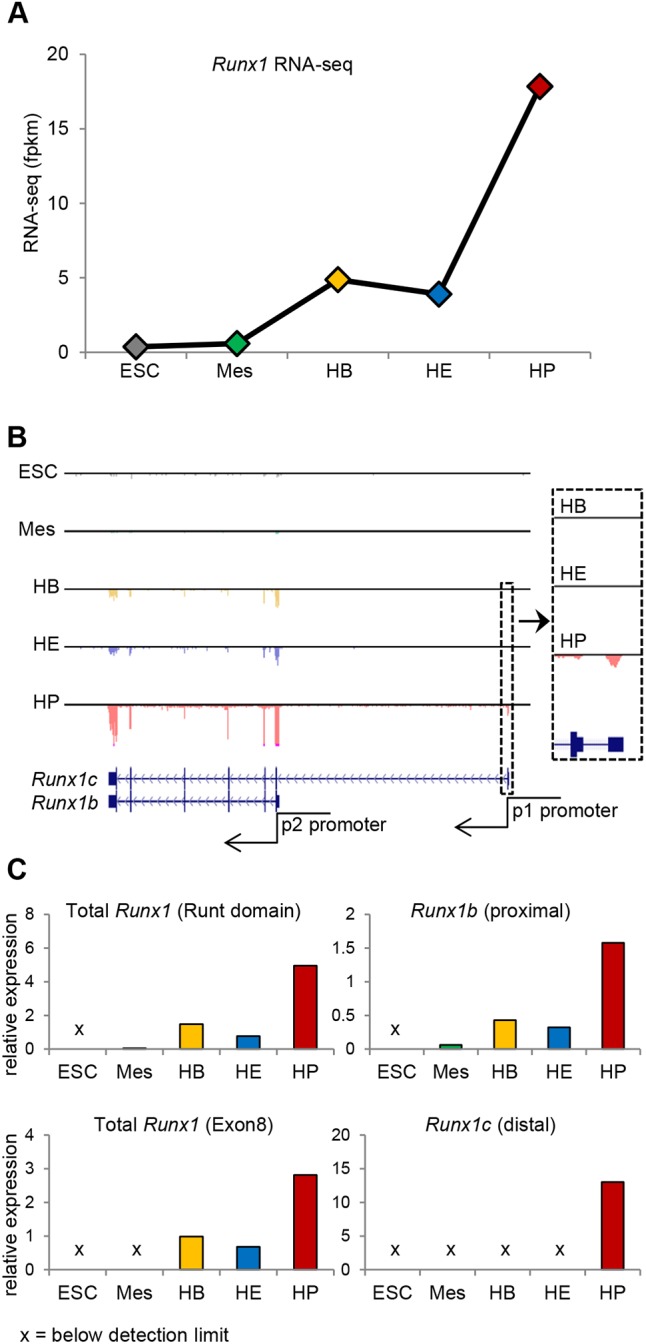
**Biphasic *Runx1* expression during hematopoietic differentiation of mESCs.** (A) *Runx1* RNA expression determined by RNA-seq across five stages of differentiation. Fpkm, fragments/kilobase of transcript/million mapped reads. (B) Mouse *Runx1* locus depicting *Runx1b* (P2-promoter driven) and *Runx1c* (P1 promoter driven) with mapped RNA-seq data. (C) qPCR across five stages of differentiation. Runt domain and exon 8 primers detect both *Runx1b* and *Runx1c* transcripts. Mean of *n*=5 or *n*=6 technical replicates is shown. Cross indicates below detection limit.

Upon mESC differentiation as embryoid bodies (EBs), the differential activity of the two *Runx1* promoters results in sequential expression of *Runx1b* and *Runx1c* (Sroczynska et al., 2009a). Proximal *Runx1b* transcripts are detected first during the early stage of the EB culture whereas distal *Runx1c* transcripts are only detected at later stages. To determine the transcript levels of *Runx1b* and *Runx1c* at key developmental stages of blood formation, we queried our recently published genome-scale resource encompassing enriched mesoderm (Mes), HB, HE and HP cell populations (Goode et al., 2016; Lancrin et al., 2010). Analysis of the RNA-seq datasets revealed that *Runx1* has a biphasic expression pattern across the different populations (Fig. 1A,B). Relatively low expression of *Runx1* in HBs and HE is followed by a 4.6-fold increase in HPs (Fig. 1A,B). Furthermore, only the *Runx1b* isoform, generated from the P2 promoter, was detected in HB and HE cells whereas both *Runx1b* and *Runx1c* were present in HPs (Fig. 1B). The biphasic pattern of expression and the specific expression of the *Runx1b* isoform were confirmed by qPCR (Fig. 1C). These data indicate that the initiation of EHT is associated with relatively low levels of *Runx1b* transcripts.

### High *Runx1* induction in iRunx1ko induces accelerated EHT

**Fig. 2.**
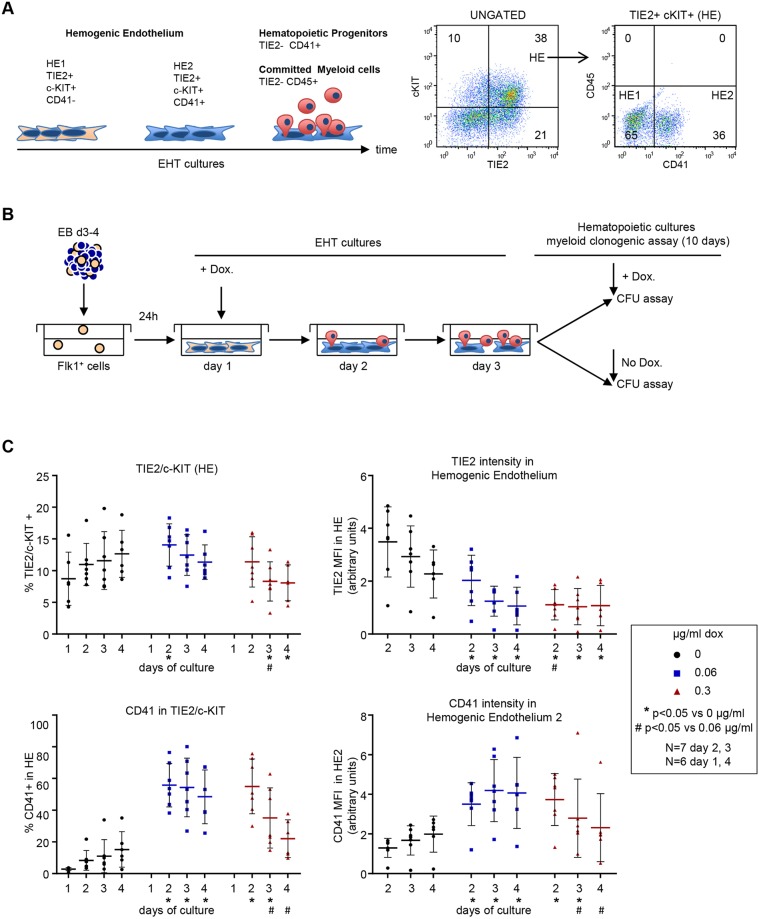
**High *Runx1* induction in iRunx1ko induces accelerated EHT.** (A) Left: schematic of hemogenic endothelium differentiation in mESC-derived EHT cultures. HE1 (TIE2+/c-KIT+/CD41−) cells transition via the HE2 stage (TIE2+/c-KIT+/CD41+) into TIE2−/CD41+ hematopoietic progenitors and TIE2−/CD45+ committed myeloid cells. Right: flow cytometry of HE1 and HE2 populations in day 2 wild-type mESC-derived EHT cultures. (B) Schematic of hematopoietic differentiation experiments. FLK1^+^ cells, isolated from mESCs differentiated as EBs, were induced with doxycycline 1 day after re-plating in EHT-culture conditions. Day 2 and 3 EHT cultures were subjected to hematopoietic CFU assays with or without additional doxycycline. (C) Flow cytometry data of a 4-day time course depicting the transition through HE1 and HE2 in iRunx1ko cells after induction by low (0.06 µg/ml) and high (0.3 µg/ml) levels of doxycycline. Top left: percentage HE cells. Top right: median fluorescence intensity of TIE2+ cells in HE. Bottom left: percentage of HE2 cells within HE. Bottom right: median fluorescence intensity of CD41+ cells within HE2. Individual biological experiments and mean±s.d. are shown. Two-way ANOVA. *N*=biological replicates.

HE generated from mESC differentiation is defined as a cell population that is positive for the endothelial markers TIE2 (TEK) or CDH5 and the stem cell growth factor receptor c-KIT (Fig. 2A, Fig. S1) (Eilken et al., 2009; Frame et al., 2016; Lancrin et al., 2009). Initially, the HE population is negative for the expression of the HP marker CD41 (termed hemogenic endothelium 1 or HE1). HE1 cells proceed to a HE2 stage by gaining cell surface expression of CD41, while retaining TIE2 and c-KIT. Subsequently, TIE2 expression is lost and HE2 cells become HP cells (TIE2^−^/CD41^+^). Most HP cells commit to myeloid hematopoiesis and acquire CD45 (PTPRC) expression. Because primitive erythropoiesis in the yolk sac does not depend on either RUNX1 or CBFβ (Niki et al., 1997; Okuda et al., 1996; Sasaki et al., 1996; Wang et al., 1996a,b), we focused our analyses on the generation of myeloid hematopoietic cells.

To investigate the effects of RUNX1 dosage on the dynamics of EHT and the formation of myeloid hematopoietic cells, we made use of a *Runx1*^−/−^ mESC line containing an exogenous *Runx1b* cDNA under the control of the doxycycline inducible tet-on system (iRunx1^Runx1^−/−^^, referred to as iRunx1ko hereafter) (Lancrin et al., 2009). Previous experiments have demonstrated that both myeloid and erythroid development can be rescued by doxycycline treatment of this line (Fig. S2A,B; Lancrin et al., 2009). Doxycycline titration experiments demonstrated that induction with 0.06 µg/ml and 0.3 µg/ml resulted in, respectively, low and high levels of *Runx1b* induction in iRunx1ko (Fig. S2C,D). Doxycycline titration in mESC lines in which GFP was under the control of the tet-on system demonstrated that a concentration of 0.3 µg/ml induced GFP in more than 90% of the cells (Fig. S2C). In contrast, although induction with 0.06 µg/ml still resulted in GFP expression in the majority of the cells (>70%), the mean fluorescence intensity was reduced by at least 50% when compared with the 0.3 µg/ml induction (Fig. S2C,D).

To study the effect of different levels of RUNX1B on EHT and subsequent myeloid blood cell formation, we differentiated mESCs using an EHT-culture system (Fig. 2B). One day after re-plating hemangioblast-enriched cells [FLK1 (KDR)^+^], *Runx1* transcription was induced with doxycycline. Some of these cells were re-plated into myeloid hematopoietic colony forming unit assays (CFU assay) after 2 or 3 days of EHT culture. In the EHT cultures, a clear difference (6.3-fold increase in relative density) in RUNX1B protein expression could be detected between 0.06 and 0.3 µg/ml doxycycline induction (Fig. S3A, lanes 1, 4, 7).

We first analyzed the progression of HE cells during the first 4 days of EHT culture by flow cytometry. When *Runx1b* is not induced in the iRunx1ko line, the differentiation process is blocked at the HE1 stage, as indicated by the very low frequency of HE2 (TIE2^+^/c-KIT^+^/CD41^+^) cells within the total HE (TIE2^+^/c-KIT^+^) population (Fig. 2C, left). Both low and high doxycycline induction released the iRunx1ko cultures from their HE1 arrest and resulted in the generation of HE2 cells (Fig. 2C). Both doxycycline concentrations induced a reduction in the expression of the surface marker TIE2 within the HE population (Fig. 2C, top right), further indicating that the doxycycline-induced samples were undergoing EHT. There was, however, a striking difference between the two treatments with regards to the persistence of the HE in general and of the HE2 population in particular. The high doxycycline dose resulted in an initial peak of HE2 (day 2) followed by a rapid decline. In line with this, we also observed a reduction in the cell surface levels of TIE2 (within HE) and CD41 (within HE2) over time (Fig. 2C, right). The cells induced with low doxycycline appeared to have a more gradual EHT with the HE2 cells persisting longer and the surface expression of CD41 in the HE2 remaining high throughout the 4 day culture period (Fig. 2C).

### High *Runx1* induction in iRunx1ko hampers myeloid hematopoietic potential

**Fig. 3.**
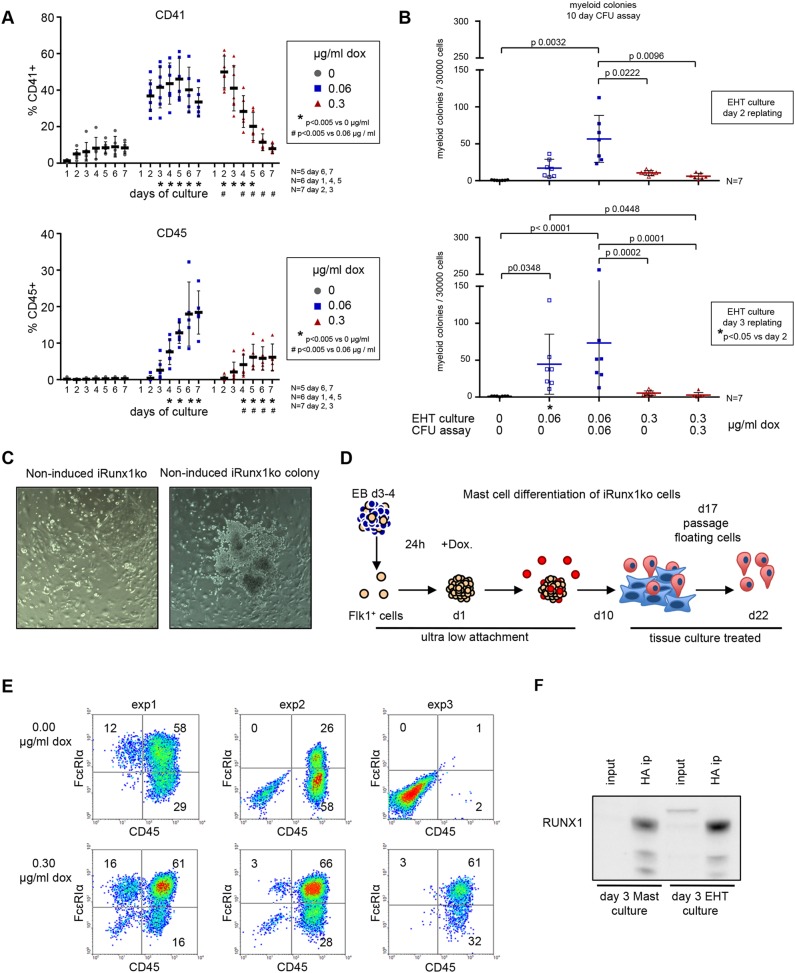
**High *Runx1* induction in iRunx1ko hampers hematopoietic potential.** (A) Flow cytometry time course depicting the percentage of CD41+ (top) and CD45+ (bottom) cells in iRunx1ko upon doxycycline induction. Individual biological experiments and mean±s.d. are shown. Two-way ANOVA. *N*=biological replicates. (B) Myeloid hematopoietic colony-formation assay with iRunx1ko re-plated after 2 (top) or 3 (bottom) days of EHT culture. Individual biological experiments and mean±s.d. are shown. Two-way ANOVA. *N*=biological replicates. (C) iRunx1ko day 3 EHT-cultures cultured for 5 days in liquid hematopoietic mix. Right-hand panel shows a rare colony of budding cells in non-induced iRunx1ko cells. (D) Schematic of mast cell differentiation. FLK1+ cells were re-plated in mast cell media under ultra-low-attachment conditions. One day after re-plating, the cells were induced with doxycycline. Mast assays were analyzed after 22 days of culture. (E) Flow cytometry analysis of three independent iRunx1ko mast assays with or without doxycycline. In two out of three experiments, CD45+ cells could be generated in the absence of doxycycline induction. (F) RUNX1 western blot on day 3 non-induced iRunx1ko mast and EHT culture HA-tag immunoprecipitations. Input represents 5% of the total lysates. HA antibody raised in mouse, RUNX1 antibody raised in rabbit.

To determine whether the seemingly rapid transition of iRunx1ko through EHT initiated by the high doxycycline induction resulted in an accelerated and/or an increased myeloid hematopoietic potential, we followed CD41 (marking HE2 and HP cells) and CD45 (marking committed myeloid cells) expression over 7 days of EHT culture (Fig. 3A). Initially, at day 2, we observed a higher percentage of CD41^+^ cells in the high doxycycline-induced samples compared with the low doxycycline samples. However, over time the percentage of CD41^+^ cells in the high doxycycline-induced group dropped below that of the low doxycycline-induced group (day 4 onwards) and eventually it was reduced to the amounts seen in the non-induced group (day 6-7). In contrast, in the low doxycycline-induced group the percentage of CD41^+^ cells remained relatively stable during the whole experiment. The rapid rise and fall in the CD41^+^ cells in the high doxycycline-induced group did not coincide with a faster conversion into CD45^+^ hematopoietic cells nor did it result in an increase in the percentage of CD45^+^ cells compared with the low doxycycline-induced group (Fig. 3A). In fact, the low doxycycline-induced group was more efficient at generating CD45^+^ cells at any time point during the culture (Fig. 3A). We also performed myeloid CFU assays on cells from day 2 and day 3 EHT cultures (Fig. 3B). Only low doxycycline efficiently generated myeloid colonies whereas high doxycycline-induced cells were severely impaired in their ability to generate myeloid colonies (Fig. 3B).

Together, these results suggest that EHT cultures expressing high RUNX1B levels rapidly transit through HE and EHT. However, the resulting cells are not able to generate myeloid colonies efficiently. In contrast, lower levels of RUNX1B resulted in a more gradual EHT and the efficient generation of myeloid progenitors.

### Leaky expression of RUNX1B in iRunx1ko drives low frequency hematopoiesis

Cells from non-induced day 3 iRunx1ko EHT cultures did not generate hematopoietic colonies in CFU assays (Fig. 3B). However, when these cells were cultured in liquid hematopoiesis-supporting media for 5 days, we observed occasional patches of cells resembling the round hematopoietic cells generated during EHT (Fig. 3C). These patches were very rare, possibly derived from the low frequency CD41^+^ cells observed in the non-induced EHT cultures (Fig. 3A). To assess this low hematopoietic potential further, we re-aggregated FLK1^+^ iRunx1ko cells under culture conditions favoring the generation of mast cells (Fig. 3D). Although this mast assay is not quantitative, the highly proliferative nature of the mast cells combined with an extended 3 weeks of culture makes it a very sensitive detection system for a low frequency of hematopoietic potential. In two out of three independent differentiations, non-induced iRunx1ko cells were able to produce phenotypic mast cells characterized by expression of CD45 and the mature mast cell marker FcεRIα (Fig. 3E). Upon doxycycline induction, mast cell generation was more efficient and was observed in all experiments (Fig. 3E). We verified that generation of hematopoietic CD45^+^ cells in this assay is dependent on the presence of intact *Runx1* by performing lentiviral rescue experiments on *Runx1^−/−^* mESCs (Fig. S4). Mast cell production could only be rescued by lentiviral transduction with a wild-type *Runx1b* cDNA and not by mutated or truncated *Runx1b* cDNAs. Immunoprecipitation experiments (utilizing the HA tag in the iRunx1ko *Runx1b* cDNA and performed with large amounts of cells) confirmed the presence of low levels of RUNX1 in non-induced iRunx1ko mast and EHT cultures (Fig. 3F). These data indicate that under appropriate culture conditions the leaky activity of the non-induced tet-on system is capable of initiating EHT, although at very low frequency, suggesting that low levels of RUNX1B could be sufficient to initiate this process.

### *Cbfb^−/−^* have reduced RUNX1 protein levels and cannot initiate EHT

The results of the mast assays prompted us to investigate the role of RUNX1B concentrations lower than those generated with 0.06 µg/ml doxycycline. However, lowering the concentration of doxycycline below 0.06 µg/ml rendered a large proportion of the cells unresponsive (Fig. S2C). To investigate a lower range of RUNX1B levels and activity we decided to target the RUNX1 binding partner CBFβ. Heterodimerization of RUNX1 with CBFβ has been shown to enhance RUNX1 DNA-binding capacity and to protect it from degradation (Huang et al., 2001; Nagata et al., 1999; Ogawa et al., 1993; Tang et al., 2000). To confirm that CBFβ interacts with RUNX1 in our EHT cultures and could potentially enhance its DNA-binding capacity, we performed Duolink proximity ligation assays for RUNX1 and CBFβ in HE1, HE2 and HP cells (Fig. S5A,B). Interactions between endogenous RUNX1 and CBFβ, detected as individual puncta, were abundant in the nuclei of all populations analyzed. This suggests that the enhancement of RUNX1 DNA-binding capacity that has been shown to result from heterodimerization with CBFβ (Nagata et al., 1999; Ogawa et al., 1993; Tang et al., 2000) could also occur within our culture system. We therefore proceeded to disrupt *Cbfb* using CRISPR/Cas9 targeting exon 4, which is part of the RUNX1-binding domain of CBFβ (Kagoshima et al., 1996; Ogawa et al., 1993). The resulting *Cbfb* knockout cells are not only expected to contain less RUNX1 protein but the remaining RUNX1 protein should be less efficient at activating RUNX1 target genes.

**Fig. 4.**
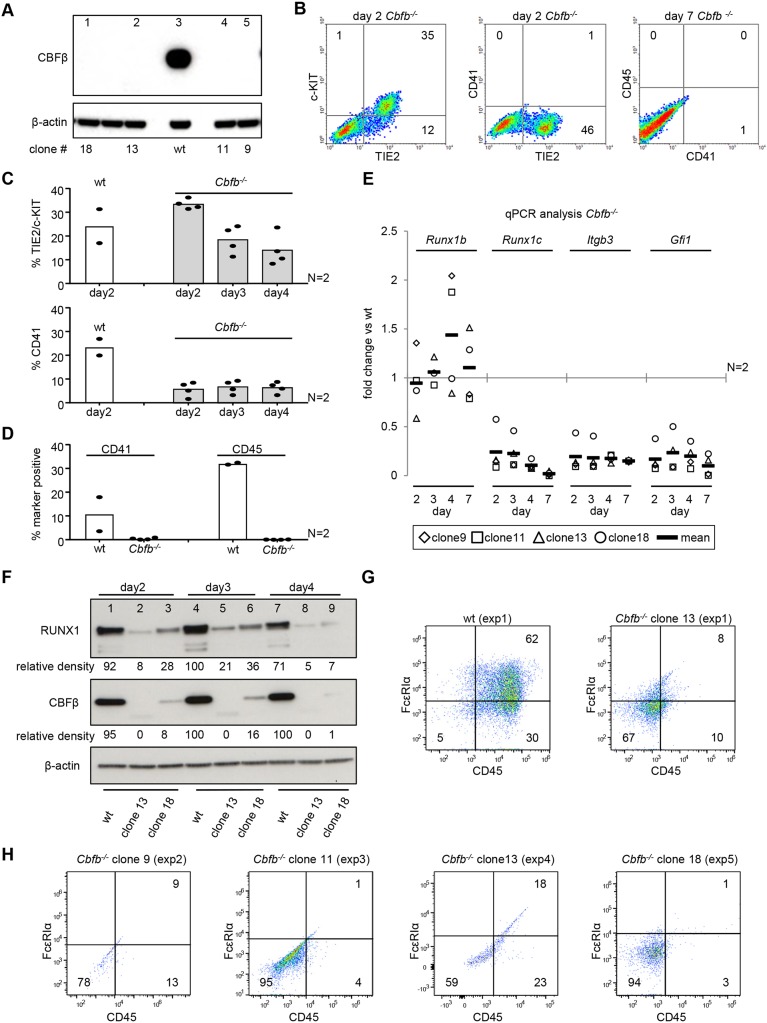
***Cbfb*^−/−^ have reduced RUNX1 protein levels and cannot initiate EHT.** (A) Western blot for CBFβ on CRISPR/Cas9-generated *Cbfb^−/−^* mESC clones. Lane 3 shows a wild-type (wt) mESC positive control. (B) Representative flow cytometry plots for *Cbfb^−/−^* day 2 and day 7 EHT cultures. (C-E) Four *Cbfb^−/−^* clones were differentiated across two independent experiments. Individual clones and mean are shown. (C,D) Flow cytometry data for four *Cbfb^−/−^* clones analyzed on day 2-4 (C) and day 7 (D) of EHT culture. (E) Time-course qPCR analysis of *Cbfb*^−/−^ EHT cultures. Data are normalized to the wild-type line. (F) Time-course western blot analysis of EHT cultures for RUNX1, CBFβ and β-actin on two *Cbfb*^−/−^ clones (lanes 2, 5, 8, and 3, 6, 9) and wild type (lanes 1, 4, 7). β-actin-normalized relative densities for RUNX1 and CBFβ are shown. (G,H) Flow cytometry analysis of four *Cbfb^−/−^* clones across five independent mast cell differentiations.

Following CRISPR/Cas9 targeting of *Cbfb* in wild-type mESCs, knockout clones were identified by the absence of detectable CBFβ protein on western blot (Fig. 4A). To analyze the effect of *Cbfb* deletion on EHT and hematopoietic development, four different *Cbfb^−/−^* mESC clones were differentiated once across two biological experiments. Flow cytometry analysis of the EHT cultures indicated that, similarly to the non-induced iRunx1ko cells, the *Cbfb^−/−^* lines fail to proceed beyond the HE1 stage (Fig. 4B,C). The *Cbfb^−/−^* lines also failed to generate CD45^+^ mature hematopoietic cells after 7 days of EHT culture (Fig. 4B,D). This block in hematopoiesis was observed despite the fact that *Runx1b* transcript levels in these *Cbfb^−/−^* lines were similar to those observed in the wild-type parent line (Fig. 4E). By day 4, two out of four clones displayed a two-fold increase in *Runx1b* RNA (suggestive of transcriptional compensation or an accumulation of HE1 cells) whereas the other clones had similar levels of *Runx1b* RNA as the parent line. Additional qPCR further confirmed a defect in the ability of *Cbfb^−/−^* cells to undergo EHT as only minimal expression of the RUNX1B early target genes *Gfi1* and *Itgb3* were detected as well as minimal expression of *Runx1c* transcripts, which are indicative of HP formation (Fig. 4E).

As shown for two *Cbfb^−/−^* clones (13 and 18), RUNX1 protein levels were reduced at all time points analyzed (Fig. 4F). In clone 18, a residual level of CBFβ was observed suggesting that some of the CRISPR/Cas9-generated indels did not completely abrogate CBFβ production. The reduction of RUNX1 protein in the *Cbfb^−/−^* lines suggests that in the EHT cultures RUNX1 is no longer protected by CBFβ from proteasomal degradation, as has been described in other culture systems (Huang et al., 2001). This is further supported by our finding that the reduction in RUNX1 could, at least partially, be rescued by treating the *Cbfb^−/−^* EHT cultures with the proteasome inhibitor MG132 (Fig. S5C). To confirm that the *Cbfb^−/−^* cells had lost their ability to initiate EHT, we performed mast assays to assess for any residual hematopoietic activity. The four *Cbfb^−/−^* clones were differentiated across five biological experiments (Fig. 4G,H) and none of them was able to generate mast cells.

Altogether, these data demonstrate that, in the absence of CBFβ, endogenous RUNX1 protein levels and activity drop below the level required to initiate EHT.

### Knockout of *Cbfb* in iRunx1ko eliminates hematopoietic potential induced by leaky RUNX1B expression

Having established that the RUNX1 levels in the *Cbfb^−/−^* lines failed to induce EHT, we next used the same CRISPR/Cas9 approach to disrupt the *Cbfb* locus in the iRunx1ko line in order to generate iRunx1ko^Cbfb^−/−^^ lines. Clones were selected by screening for the absence of detectable CBFβ protein by western blot (Fig. S6A). Induction of *Runx1b* with either 0.06 or 0.3 µg/ml doxycycline resulted in similar *Runx1b* transcript levels in both the parental iRunx1ko line and the iRunx1ko^Cbfb^−/−^^ lines (Fig. S6B). However, as expected, RUNX1B protein levels were markedly lower in the iRunx1ko^Cbfb^−/−^^ lines with relative amounts reduced by more than 38% (0.3 μg/ml doxycycline) and more than 81% (0.06 μg/ml doxycycline) (Fig. S3A). Next, we tested the hematopoietic potential of the iRunx1ko^Cbfb^−/−^^ lines by performing mast assays (Fig. S6C). The four iRunx1ko^Cbfb^−/−^^ clones were each differentiated once across four biological experiments. None of the clones was able to generate mast cells, demonstrating that the absence of CBFβ eliminated the low hematopoietic potential observed in the non-induced iRunx1ko cells (Fig. 3E). These results demonstrate that in iRunx1ko^Cbfb^−/−^^ lower levels of RUNX1B can be achieved than in the parent iRunx1ko line.

### Low-level *Runx1b* induction is sufficient to initiate EHT in iRunx1ko^Cbfb^−/−^^

**Fig. 5.**
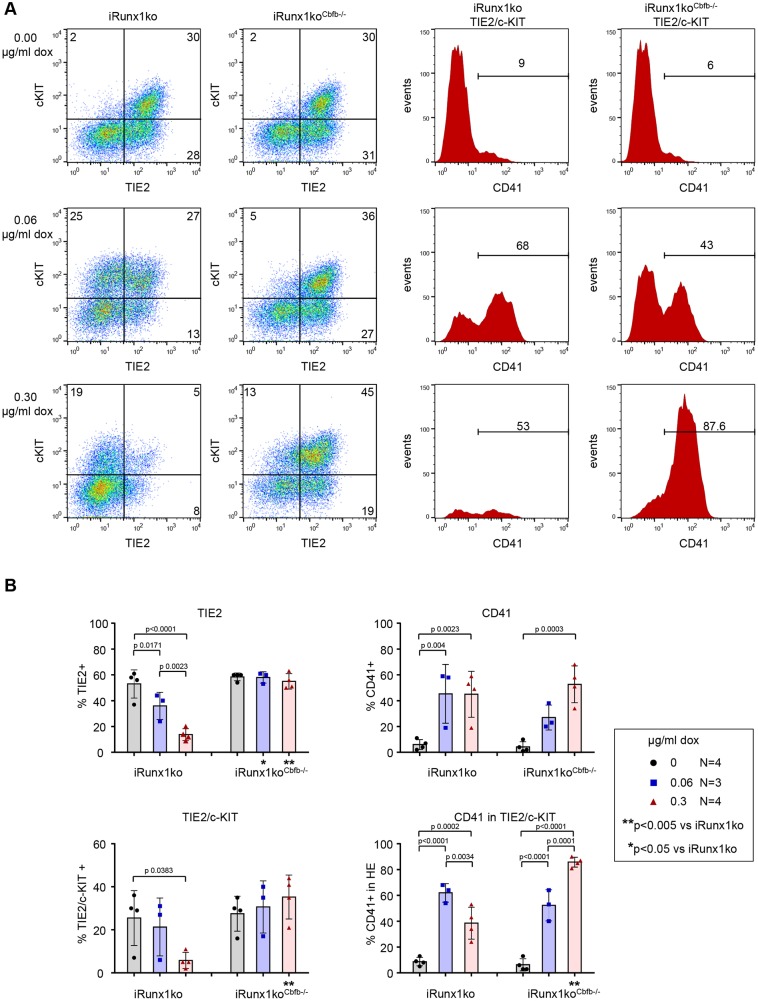
**Low RUNX1 levels can induce EHT-like changes.** (A,B) Representative flow cytometry plots (A) and flow cytometry data (B) of day 3 iRunx1ko and iRunx1ko^Cbfb^−/−^^ EHT cultures. (A) Left: HE (TIE2+/c-KIT+). Right: TIE2+/c-KIT+ gated histograms depicting the percentage CD41+ cells within HE. Horizontal bars indicate the CD41+ population. (B) Individual biological experiments and mean±s.d. are shown. Two-way ANOVA. *N*=biological replicates.

**Fig. 6.**
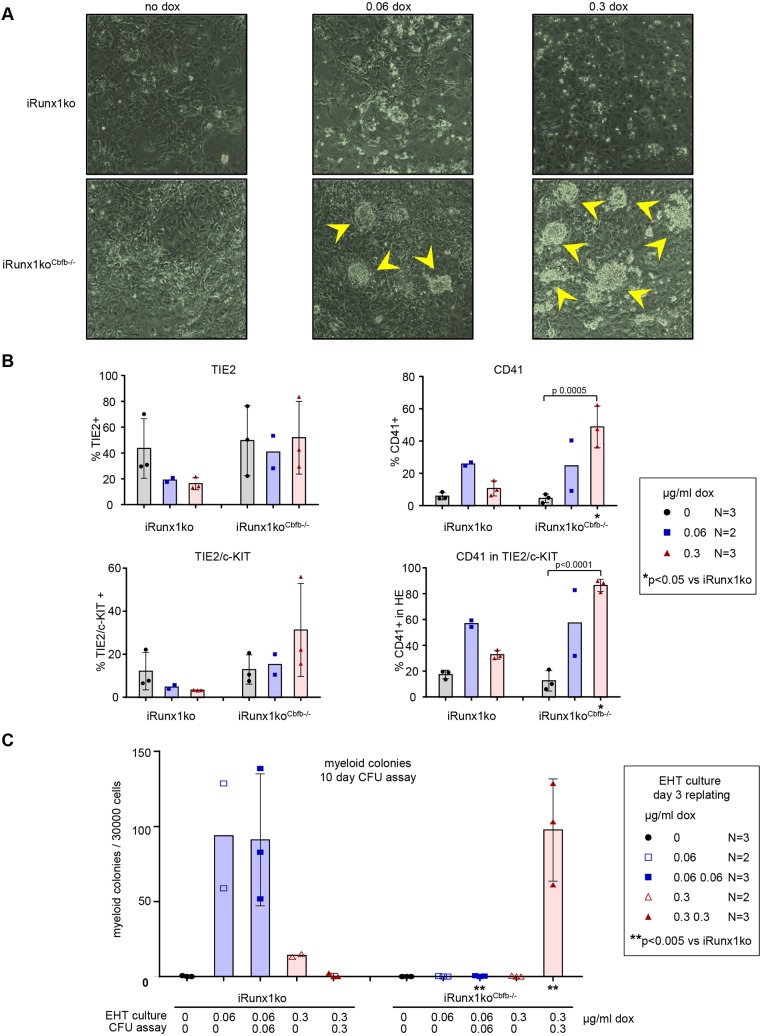
**Persistence of HE core-like structures in doxycycline-induced iRunx1ko^Cbfb^−/−^^ cultures.** (A) Representative pictures of day 5 iRunx1ko (top) and iRunx1ko^Cbfb^−/−^^ (bottom) EHT cultures. HE core-like structures (arrowheads) can be observed in doxycycline-induced iRunx1ko^Cbfb^−/−^^. (B) Flow cytometry analysis of day 5 iRunx1ko and iRunx1ko^Cbfb^−/−^^ EHT cultures with or without doxycycline. Individual biological experiments and mean±s.d. (only for *N*>2) are shown. Two-way ANOVA (only for *N*>2). (C) Myeloid hematopoietic colony-formation assay with iRunx1ko and iRunx1ko^Cbfb^−/−^^ re-plated after 3 days of EHT culture. Individual biological experiments and mean±s.d. (only for *N*>2) are shown. Two-way ANOVA (only for *N*>2). *N*=biological replicates.

We next evaluated the ability of the iRunx1ko^Cbfb^−/−^^ cells to progress through EHT as described above (Fig. 2B). Flow cytometry analysis of day 2 (Fig. S7) and 3 (Fig. 5) EHT cultures showed that upon *Runx1b* induction with either concentration of doxycycline the iRunx1ko^Cbfb^−/−^^ lines were able to progress to HE2. However, further differentiation was clearly perturbed compared with the parent iRunx1ko line (Fig. 5, Fig. S7). Upon high doxycycline induction, the percentage of CD41^+^ cells was comparable to that of the parent iRunx1ko line but the iRunx1ko^Cbfb^−/−^^ cells did not downregulate TIE2 expression as efficiently as the parent line. This was reflected by the fact that the HE population remained high in the induced iRunx1ko^Cbfb^−/−^^ lines. On day 3, nearly all of the HE population in the high doxycycline-induced iRunx1^Cbfb^−/−^^ consisted of HE2 cells whereas in the identically induced parent iRunx1ko only very few HE cells remained (Fig. 5). Consistent with this, we observed, after extended culture, multiple HE core-like structures in doxycycline-induced iRunx1ko^Cbfb^−/−^^ cultures (Fig. 6A). This was particularly apparent in the high doxycycline-induced iRunx1ko^Cbfb^−/−^^ cultures and was not observed in non-induced iRunx1ko^Cbfb^−/−^^ cultures or in the original parent line (Fig. 6A). Flow cytometry revealed that the late high doxycycline-induced iRunx1ko^Cbfb^−/−^^ cultures contained high frequencies of TIE2 and CD41 cells, similar to those observed in early day 2 and 3 doxycycline-induced iRunx1ko^Cbfb^−/−^^ EHT cultures (Figs 5 and 6, Figs S7 and S9). These late cultures still retained an appreciable HE population that consisted of mainly HE2 cells (Fig. 6B, Fig. S9).

Quantitative PCR analysis confirmed the downregulation of the endothelial program in doxycycline-induced iRunx1ko EHT cultures, as indicated by reduced levels of *Cdh5*, *Sox7* and *Sox17* transcripts (Fig. S8A), which are known to be downregulated during EHT and subsequent HP formation (Clarke et al., 2013; Costa et al., 2012; Lilly et al., 2016b; Lizama et al., 2015). In contrast, the iRunx1ko^Cbfb^−/−^^ cultures showed only a modest reduction in *Sox7* and *Sox17* transcripts with high doxycycline and *Cdh5* did not decline at all (Fig. S8A). We also determined the levels of early hematopoietic target genes, such as *Itgb3* (CD61) and *Gfi1*, which are both early RUNX1 targets and mark the early stages of EHT (Lancrin et al., 2012; Lie-A-Ling et al., 2014; Thambyrajah et al., 2016), and *Gfi1b* and *AI467606* (SAIL), which mark the next progenitor stage (Ferreras et al., 2011; Kim et al., 2015; Thambyrajah et al., 2016). These early hematopoietic target genes were activated to a similar level by both doses of doxycycline in iRunx1ko cultures (Fig. S8B). In contrast, in the iRunx1ko^Cbfb^−/−^^ lines *Itgb3*, *Gfi1* and *AI467606* transcripts were significantly induced only by high doxycycline induction (Fig. S8B). Altogether, these results suggest that the lower levels of RUNX1 protein and activity achieved in the induced iRunx1ko^Cbfb^−/−^^ lines (compared with the iRunx1ko parent line) are sufficient to progress to the HE2 stage but that higher doses/activity of RUNX1 are required for the generation of hematopoietic progenitors.

### Myeloid hematopoiesis in iRunx1ko^Cbfb^−/−^^ requires continuous high-level *Runx1b* induction

To investigate further the RUNX1B levels required to generate hematopoietic cells in the iRunx1ko^Cbfb^−/−^^ clones, we performed myeloid CFU assays following EHT culture (Fig. 2B). These experiments revealed that a single dose of doxycycline at day 1 of the EHT culture did not allow the iRunx1ko^Cbfb^−/−^^ cells to generate hematopoietic colonies (Fig. 6C). This is consistent with our findings that these cells are blocked at the HE2 stage (Figs 4 and 5). This result was further corroborated by the observation that a single dose of doxycycline applied at day 1 of iRunx1ko^Cbfb^−/−^^ mast assays also failed to produce hematopoietic cells (Fig. S6C). However, with an additional high dose of doxycycline at the start of the myeloid hematopoietic CFU assay we found that iRunx1ko^Cbfb^−/−^^ cells generated myeloid hematopoietic colonies (Fig. 6C). This is in contrast to the parent iRunx1ko line, in which a single dose of low doxycycline at day 1 was sufficient for colony formation whereas high doxycycline abolished most colony-formation capability (Fig. 6C).

Altogether, these results show that the reduced levels of RUNX1B protein and activity obtained upon a single dose of doxycycline induction of the iRunx1ko^Cbfb^−/−^^ lines is sufficient to initiate EHT. However, the cells arrest at the HE2 stage and are not able to complete EHT. This process can only be completed, and hematopoiesis rescued, by treating the HE2-arrested cells with an additional high dose of doxycycline. This indicates that a critical level of RUNX1B activity is required not only to initiate but also to complete EHT and suggests that this critical level might be different for initiation and completion of EHT. These data also establish that enforcing the expression of RUNX1B rescues the block of hematopoietic development observed in the absence of CBFβ.

### The CBFβ competitor SOX7 can bind RUNX1 in mESC-derived HE

Our findings indicate that different levels and activities of RUNX1 are crucial at different stages of EHT. This is likely to be achieved by a combination of regulation of the transcription of *Runx1b* as well as modulation of RUNX1 activity by interactions with other proteins and post-translational modifications. In this context, it is interesting that the SOX7 transcription factor has been recently shown to regulate hematopoietic RUNX1 activity at a protein level by directly competing with CBFβ (Lilly et al., 2016a). To determine whether such a mechanism could also play a role in the control of RUNX1 activity in our mESC-derived EHT-culture system, we queried previously generated RNA-seq datasets (Goode et al., 2016) and performed qPCR. We observed that *Sox7* is preferentially expressed in the HE population (Fig. S10A). In addition, Duolink proximity ligation assays demonstrated that interactions between endogenous RUNX1 and SOX7 were abundant in the nuclei of mESC-derived HE cells (Fig. S10B). These findings suggest that SOX7 might control hematopoietic RUNX1 activity in mESC-derived HE by counteracting CBFβ-mediated enhancement of RUNX1 DNA binding and/or protection from degradation. To investigate the mechanism of RUNX1 regulation by SOX7 further, we introduced an additional doxycycline-inducible *Sox7* expression cassette (tet-on system) into two iRunx1ko^Cbfb^−/−^^ clones (iRunx1ko_Sox7^Cbfb^−/−^^). In iRunx1ko_Sox7^Cbfb^−/−^^ mESCs, both *Runx1b* and *Sox7* could be induced by the addition of doxycycline (Fig. S10C,D). Both iRunx1ko^Cbfb^−/−^^ and iRunx1ko_Sox7^Cbfb^−/−^^ mESCs produced similar amounts of *Runx1b* transcripts upon doxycycline induction (Fig. S10C,D). However, RUNX1B protein levels were consistently higher in the induced iRunx1ko_Sox7^Cbfb^−/−^^ mESCs (Fig. S10C,D). This suggests that in the HE populations SOX7 might modulate RUNX1 activity by protecting it from degradation and sequestering it from its activator, CBFβ. Such a mechanism could allow for rapid and precise control of RUNX1 activity within the HE population.

## DISCUSSION

RUNX1 is a master regulator of hematopoiesis, which is indispensable for the establishment of the hematopoietic system during embryogenesis (Chen et al., 2009; Lacaud et al., 2002; North et al., 1999; Okuda et al., 1996). Although RUNX1 is not essential for survival once the adult blood system has been established, its absence or the reduction of its level still results in perturbations in the specification and differentiation of multiple blood lineages (Chen et al., 2009; Growney et al., 2005; Ichikawa et al., 2004). Similarly, during ontogeny *Runx1* haploinsufficiency or mutations result in decreased numbers of hematopoietic stem and/or progenitor cells (Cai et al., 2000; Lacaud et al., 2002, 2004; Matheny et al., 2007; Wang et al., 1996a). However, the specific RUNX1 dosage requirements in HE and during different stages of EHT remain largely unknown.

By combining transcriptional control with the modulation of RUNX1 stability and activity by deleting *Cbfb*, we demonstrate that RUNX1 is required for both the initiation and completion of EHT. Furthermore, at the onset of hematopoiesis RUNX1 dosage must be carefully controlled in HE. A high dose of RUNX1B rapidly induced a post-EHT phenotype but the resulting cells had only minor hematopoietic potential. In contrast, a lower dose of RUNX1B allowed for a more gradual EHT induction. On a *Cbfb* wild-type background, a temporally restricted low dose of RUNX1B at the initiation of the EHT is sufficient to proceed through EHT fully and produce hematopoietic colonies. However, when RUNX1 stability and activity was perturbed (by disrupting the *Cbfb* locus), continuous high-level *Runx1b* induction was required to rescue hematopoiesis. Interestingly, when *Runx1b* was temporarily induced in a *Cbfb*^^−/−^^ background, using either low or high *Runx1b* induction, the cells were able to progress from the HE1 stage to the HE2 stage. This suggests that although both the initiation of EHT and the subsequent generation of hematopoietic precursors require RUNX1 activity the required threshold of RUNX1 activity might be different for EHT initiation and completion.

A recent study suggests that in zebrafish Runx1 can drive the generation of hematopoietic stem cells (HSCs) in the absence of Cbfβ (Bresciani et al., 2014), implying that Runx1 can initiate EHT independently of Cbfβ. In contrast, in mice *Cbfb* knockouts display a similar phenotype as *Runx1* knockouts (Niki et al., 1997; Okuda et al., 1996; Sasaki et al., 1996; Wang et al., 1996a,b). However, it is unclear whether in *Cbfb*^^−/−^^ mice EHT changes can be initiated by residual RUNX1 activity. Our *in vitro* results suggest that this is probably not the case. During the differentiation of *Cbfb*^^−/−^^ mESCs, we did not detect robust expression of the early HE RUNX1 target genes *Itgb3* and *Gfi1*. Furthermore, the cells did not activate appreciable *Runx1c* transcription and did not generate CD41^+^ HP or CD45^+^ hematopoietic cells. This difference with the phenotype observed in zebrafish could be due to different requirements for RUNX1 activity between the two species. Indeed, it has been reported that in zebrafish adult HSCs can be formed without wild-type *Runx1* (Sood et al., 2010).

Using sustained high-level induction of *Runx1b*, we were able to rescue myeloid hematopoiesis in *Cbfb^^−/−^^* cells. This indicates that if RUNX1 levels are sufficiently high, it can function independently of CBFβ. However, a previous study by Yokomizo et al. performed in *Cbfb*^^−/−^^ mice demonstrated, in contrast, that *Runx1* overexpression was not able to restore hematopoiesis (Yokomizo et al., 2008). These contrasting findings could be due to the use, by Yokomizo et al., of a GATA-1 gene regulatory cassette (GATA-1-HRD) to overexpress *Runx1*. The most robust activity of this cassette is restricted to erythroid progenitors (Yoon et al., 2008). In addition, in our hands *Runx1* overexpression only rescued hematopoiesis in *Cbfb*^^−/−^^ cells if overexpression was maintained throughout the EHT and subsequent differentiation steps. Furthermore, we found that temporal pre-EHT *Runx1* expression can initiate EHT, or at least promote similar early changes, but is not sufficient to complete the process in the absence of CBFβ. Thus, the absence of hematopoietic rescue when using the GATA-1-HRD cassette to express *Runx1* in the context of a *Cbfb*^^−/−^^ background might be explained by insufficient levels of *Runx1* transcription in cell types relevant to the onset of hematopoiesis. Our data, in concordance with previous reports, show that under normal physiological conditions CBFβ is essential for hematopoiesis and reveals that, in the mESC system, CBFβ is required at the very onset of EHT.

The RUNX1B dosage experiments also highlight the need for relatively moderate levels of RUNX1B activity in order to initiate a productive EHT. This suggests that RUNX1B dosage or activity must be tightly controlled in pre-hematopoietic progenitor populations, in particular in the HE. Quantification of *Runx1* transcripts suggests that transcriptional control and/or mRNA turnover plays a role in determining RUNX1 dosage at the early stages of differentiation (this study; Swiers et al., 2013). However, given the transient nature of the HE and the dynamic nature of EHT, dependence on transcriptional control alone is unlikely. Recently, the SOX7 transcription factor has been shown to regulate hematopoietic RUNX1 activity at the protein level by directly competing with CBFβ (Lilly et al., 2016a). At the onset of hematopoiesis, *Runx1* and *Sox7* are co-expressed in the HE population (this study; Costa et al., 2012; Lilly et al., 2016a) and here we further demonstrate that endogenous SOX7 and RUNX1 can interact specifically in mESC-derived HE. We further demonstrate that SOX7 can increase RUNX1 levels independently of transcription in the absence of CBFβ. Interestingly, SOX7 overexpression in pre-EHT populations has been shown to cause accumulation of cells in HE2 both in mESC-derived cells and in mouse yolk sac explant cultures (Costa et al., 2012; Lilly et al., 2016a). This phenotype is similar to the HE2 block we observed when we induced *Runx1b* expression on a *Cbfb^^−/−^^* background. It would be therefore interesting to determine whether some of the early RUNX1 target genes in HE are in part controlled by the RUNX1-SOX7 complexes. However, although the binding sites of RUNX1B in mouse ESC-derived HE have been mapped (Lie-A-Ling et al., 2014), this has not yet been achieved for SOX7. However, our finding underscores the intriguing possibility that interaction of RUNX1 with proteins other than CBFβ plays a role in regulating or re-directing RUNX1 activity in HE and EHT. Further studies will be required to elucidate whether such mechanisms are in place and to what extent the RUNX1-SOX7 complex can participate.

The data presented here suggests a model in which RUNX1 is crucial for the initiation and completion of EHT and the regulation of RUNX1B dosage in HE is crucial for the efficient progression of EHT and blood formation (Fig. S11). In early HE (HE1), RUNX1B protein is present, but its activity might be minimal. This facilitates HE maturation and the initiation and progression of EHT. Circumventing this HE maturation phase, by increasing RUNX1B levels too early, results in abortive hematopoiesis. Once EHT is initiated RUNX1 activity is still required in order to produce mature hematopoietic cells. This process might be facilitated by modulating RUNX1 activity, potentially by changing RUNX1 binding partners, and also later by increasing *Runx1* transcription and switching from the *Runx1b* to the *Runx1c* isoform.

In summary, this study reveals that sustained RUNX1 activity is required from the initiation to the completion of the EHT and indicates that RUNX1 transcription and activity need to be strictly controlled during this process.

## MATERIALS AND METHODS

### Mouse ESC lines and culture

The following ESC lines were used: Ainv18 (wild-type ESC), *Runx1^^−/−^^*, doxycycline-inducible line iRunx^Runx1^−/−^^ (iRunx1ko), Sox7-GFP (Gandillet et al., 2009; Kyba et al., 2002; Lancrin et al., 2009). All lines were authenticated and regularly checked for mycoplasma contamination. ESCs were maintained and differentiated as previously described (Sroczynska et al., 2009b). Briefly, hemangioblast-enriched cell populations isolated from day 3.5 EBs by magnetic-activated cell sorting for FLK1 (Miltenyi Biotec) (Table S1) were cultured in liquid hemangioblast colony-forming media (EHT cultures). Hematopoietic assays and hematopoietic colony-forming assays were performed by transferring EHT cultures to liquid or semisolid hematopoietic mix. For mast-assays, hemangioblast-enriched cells were aggregated in EB medium with IL3, KIT ligand (both 0.5% conditioned media) and IL6 (5 ng/ml) in ultra-low attachment plates (CoStar) as previously described (Fehling et al., 2003). After 10 days, cells were transferred to tissue-culture-treated dishes and cultured for 7 days. Non-attached cells were passaged and cultured for 5 days. All tissue culture images were taken on a DMI 3000B with a DFC310 FX camera (Leica).

### CRISPR-Cas9 *Cbfb* knockout

*Cbfb* CRISPR knockout lines were generated with D10A mutant Cas9 nickase (Cas9n) (Addgene plasmid #48140, deposited by Feng Zhang), as previously described (Ran et al., 2013). Primers used to generate guides, targeting exon 4 of mouse *Cbfb*, are provided in Table S2.

### Quantitative reverse transcription PCR

RNA was extracted using the RNeasy Micro Kit (Qiagen). For the confirmation of RNA-seq data, RNA was reverse transcribed with random hexamer oligos and SuperScriptIII First Strand Synthesis (Invitrogen). To maximize the limited quantity of sample, cDNA was run on a 96.96 Array (Fluidigm) on the BioMark HD platform according to the manufacturer’s protocol. For all other samples, cDNA was prepared with Omniscript RT (Qiagen) and random hexamer oligos. Real-time PCR was performed using the TaqMan assay (Applied Biosystems) and Universal Probe Library (Roche) and run on an ABI 7900. Primer sequences are listed in Table S2.

### Flow cytometry

Antibodies are listed in Table S1. Cells dissociated by trypsinisation were analyzed by flow cytometry (Table S3; BD Biosciences/ACEA Biosciences).

### Western blot/immunoprecipitation

Total proteins were extracted with the Nuclear Extract Kit (Active Motif) according to the manufacturer’s protocol. Lysates were subjected to SDS-PAGE and transferred to PVDF membranes. Immunoprecipitations were performed with EZview Red Anti-HA Affinity Gel (Sigma-Aldrich) according to the manufacturer’s protocol. Antibodies are listed in Table S1.

### Duolink proximity ligation assay

The Duolink assay was performed and analyzed as described previously (Lilly et al., 2016a). Antibodies are listed in Table S1. Images were taken using a Zeiss Axiovert 200M microscope at 40× or 100× magnification and analyzed using ImageJ software.

### *Runx1b* lentiviral vectors

Lentiviral vectors with murine *Runx1b* cDNA were produced as described previously (Dull et al., 1998; Seppen et al., 2000; Zufferey et al., 1998). Mutations were introduced with QuikChangeII Site-Directed Mutagenesis Kit (Agilent Technologies). Transduction of FLK1^+^ cells was performed as previously described (Gandillet et al., 2009). For details, see supplementary Materials and Methods.

### Inducible *Sox7* PiggyBAC transposon

Inducible *Sox7* transposon-containing mESCs were established by PEI co-transfection with PiggyBAC transposase (Bartman et al., 2015). Lines were established by magnetic-activated cell sorting for thNGF (Table S1). For details, see supplementary Materials and Methods.

### Statistics

Two-way ANOVA was used to assess differences (GraphPad Prism 7).

## Supplementary Material

Supplementary information

Supplementary information

## References

[DEV149419C1] Banno K., Omori S., Hirata K., Nawa N., Nakagawa N., Nishimura K., Ohtaka M., Nakanishi M., Sakuma T., Yamamoto T. et al. (2016). Systematic cellular disease models reveal synergistic interaction of trisomy 21 and GATA1 mutations in hematopoietic abnormalities. *Cell Rep.* 15, 1228-1241. 10.1016/j.celrep.2016.04.031 27134169

[DEV149419C2] Bartman C. M., Egelston J., Ren X., Das R. and Phiel C. J. (2015). A simple and efficient method for transfecting mouse embryonic stem cells using polyethylenimine. *Exp. Cell Res.* 330, 178-185. 10.1016/j.yexcr.2014.07.020 25102378 PMC4268182

[DEV149419C3] Bee T., Liddiard K., Swiers G., Bickley S. R. B., Vink C. S., Jarratt A., Hughes J. R., Medvinsky A. and de Bruijn M. F. T. R. (2009). Alternative Runx1 promoter usage in mouse developmental hematopoiesis. *Blood Cells Mol. Dis.* 43, 35-42. 10.1016/j.bcmd.2009.03.011 19464215

[DEV149419C4] Bee T., Swiers G., Muroi S., Pozner A., Nottingham W., Santos A. C., Li P. S., Taniuchi I. and de Bruijn M. F. T. R. (2010). Nonredundant roles for Runx1 alternative promoters reflect their activity at discrete stages of developmental hematopoiesis. *Blood* 115, 3042-3050. 10.1182/blood-2009-08-238626 20139099

[DEV149419C5] Ben-Ami O., Friedman D., Leshkowitz D., Goldenberg D., Orlovsky K., Pencovich N., Lotem J., Tanay A. and Groner Y. (2013). Addiction of t(8;21) and inv(16) acute myeloid leukemia to native RUNX1. *Cell Rep.* 4, 1131-1143. 10.1016/j.celrep.2013.08.020 24055056

[DEV149419C6] Bertrand J. Y., Chi N. C., Santoso B., Teng S., Stainier D. Y. R. and Traver D. (2010). Haematopoietic stem cells derive directly from aortic endothelium during development. *Nature* 464, 108-111. 10.1038/nature08738 20154733 PMC2858358

[DEV149419C7] Boisset J.-C., van Cappellen W., Andrieu-Soler C., Galjart N., Dzierzak E. and Robin C. (2010). In vivo imaging of haematopoietic cells emerging from the mouse aortic endothelium. *Nature* 464, 116-120. 10.1038/nature08764 20154729

[DEV149419C8] Bonifer C., Levantini E., Kouskoff V. and Lacaud G. (2017). Runx1 structure and function in blood cell development. *Adv. Exp. Med. Biol.* 962, 65-81. 10.1007/978-981-10-3233-2_5 28299651

[DEV149419C9] Brady G., Elgueta Karstegl C. and Farrell P. J. (2013). Novel function of the unique N-terminal region of RUNX1c in B cell growth regulation. *Nucleic Acids Res.* 41, 1555-1568. 10.1093/nar/gks1273 23254331 PMC3561965

[DEV149419C10] Bresciani E., Carrington B., Wincovitch S., Jones M., Gore A. V., Weinstein B. M., Sood R. and Liu P. P. (2014). CBFbeta and RUNX1 are required at 2 different steps during the development of hematopoietic stem cells in zebrafish. *Blood* 124, 70-78. 10.1182/blood-2013-10-531988 24850758 PMC4125354

[DEV149419C11] Cai Z., de Bruijn M., Ma X., Dortland B., Luteijn T., Downing J. R. and Dzierzak E. (2000). Haploinsufficiency of AML1 affects the temporal and spatial generation of hematopoietic stem cells in the mouse embryo. *Immunity* 13, 423-431. 10.1016/S1074-7613(00)00042-X 11070161

[DEV149419C12] Challen G. A. and Goodell M. A. (2010). Runx1 isoforms show differential expression patterns during hematopoietic development but have similar functional effects in adult hematopoietic stem cells. *Exp. Hematol.* 38, 403-416. 10.1016/j.exphem.2010.02.011 20206228 PMC2854264

[DEV149419C13] Chen M. J., Yokomizo T., Zeigler B. M., Dzierzak E. and Speck N. A. (2009). Runx1 is required for the endothelial to haematopoietic cell transition but not thereafter. *Nature* 457, 887-891. 10.1038/nature07619 19129762 PMC2744041

[DEV149419C14] Choi K., Kennedy M., Kazarov A., Papadimitriou J. C. and Keller G. (1998). A common precursor for hematopoietic and endothelial cells. *Development* 125, 725-732. 9435292 10.1242/dev.125.4.725

[DEV149419C15] Clarke R. L., Yzaguirre A. D., Yashiro-Ohtani Y., Bondue A., Blanpain C., Pear W. S., Speck N. A. and Keller G. (2013). The expression of Sox17 identifies and regulates haemogenic endothelium. *Nat. Cell Biol.* 15, 502-510. 10.1038/ncb2724 23604320 PMC4011511

[DEV149419C16] Costa G., Mazan A., Gandillet A., Pearson S., Lacaud G. and Kouskoff V. (2012). SOX7 regulates the expression of VE-cadherin in the haemogenic endothelium at the onset of haematopoietic development. *Development* 139, 1587-1598. 10.1242/dev.071282 22492353

[DEV149419C17] De Vita S., Canzonetta C., Mulligan C., Delom F., Groet J., Baldo C., Vanes L., Dagna-Bricarelli F., Hoischen A., Veltman J. et al. (2010). Trisomic dose of several chromosome 21 genes perturbs haematopoietic stem and progenitor cell differentiation in Down's syndrome. *Oncogene* 29, 6102-6114. 10.1038/onc.2010.351 20697343 PMC3007620

[DEV149419C18] Draper J. E., Sroczynska P., Tsoulaki O., Leong H. S., Fadlullah M. Z. H., Miller C., Kouskoff V. and Lacaud G. (2016). RUNX1B expression is highly heterogeneous and distinguishes megakaryocytic and erythroid lineage fate in adult mouse hematopoiesis. *PLoS Genet.* 12, e1005814 10.1371/journal.pgen.1005814 26808730 PMC4726605

[DEV149419C19] Draper J. E., Sroczynska P., Leong H. S., Fadlullah M. Z. H., Miller C., Kouskoff V. and Lacaud G. (2017). Mouse RUNX1C regulates premegakaryocytic/erythroid output and maintains survival of megakaryocyte progenitors. *Blood* 130, 271-284. 10.1182/blood-2016-06-723635 28490570 PMC5833261

[DEV149419C20] Dull T., Zufferey R., Kelly M., Mandel R. J., Nguyen M., Trono D. and Naldini L. (1998). A third-generation lentivirus vector with a conditional packaging system. *J. Virol.* 72, 8463-8471. 9765382 10.1128/jvi.72.11.8463-8471.1998 PMC110254

[DEV149419C21] Eilken H. M., Nishikawa S.-I. and Schroeder T. (2009). Continuous single-cell imaging of blood generation from haemogenic endothelium. *Nature* 457, 896-900. 10.1038/nature07760 19212410

[DEV149419C22] Fehling H. J., Lacaud G., Kubo A., Kennedy M., Robertson S., Keller G. and Kouskoff V. (2003). Tracking mesoderm induction and its specification to the hemangioblast during embryonic stem cell differentiation. *Development* 130, 4217-4227. 10.1242/dev.00589 12874139

[DEV149419C23] Ferreras C., Lancrin C., Lie-A-Ling M., Kouskoff V. and Lacaud G. (2011). Identification and characterization of a novel transcriptional target of RUNX1/AML1 at the onset of hematopoietic development. *Blood* 118, 594-597. 10.1182/blood-2010-06-294124 21498670

[DEV149419C24] Frame J. M., Fegan K. H., Conway S. J., McGrath K. E. and Palis J. (2016). Definitive hematopoiesis in the Yolk Sac emerges from Wnt-responsive hemogenic endothelium independently of circulation and arterial identity. *Stem Cells* 34, 431-444. 10.1002/stem.2213 26418893 PMC4755868

[DEV149419C25] Fujita Y., Nishimura M., Taniwaki M., Abe T. and Okuda T. (2001). Identification of an alternatively spliced form of the mouse AML1/RUNX1 gene transcript AML1c and its expression in early hematopoietic development. *Biochem. Biophys. Res. Commun.* 281, 1248-1255. 10.1006/bbrc.2001.4513 11243869

[DEV149419C26] Gandillet A., Serrano A. G., Pearson S., Lie-A-Ling M., Lacaud G. and Kouskoff V. (2009). Sox7-sustained expression alters the balance between proliferation and differentiation of hematopoietic progenitors at the onset of blood specification. *Blood* 114, 4813-4822. 10.1182/blood-2009-06-226290 19801444

[DEV149419C27] Gilham D. E., Lie-A-Ling M., Taylor N. and Hawkins R. E. (2010). Cytokine stimulation and the choice of promoter are critical factors for the efficient transduction of mouse T cells with HIV-1 vectors. *J. Gene Med.* 12, 129-136. 20033928 10.1002/jgm.1421

[DEV149419C28] Goode D. K., Obier N., Vijayabaskar M. S., Lie-A-Ling M., Lilly A. J., Hannah R., Lichtinger M., Batta K., Florkowska M., Patel R. et al. (2016). Dynamic gene regulatory networks drive hematopoietic specification and differentiation. *Dev. Cell* 36, 572-587. 10.1016/j.devcel.2016.01.024 26923725 PMC4780867

[DEV149419C29] Goyama S., Schibler J., Cunningham L., Zhang Y., Rao Y., Nishimoto N., Nakagawa M., Olsson A., Wunderlich M., Link K. A. et al. (2013). Transcription factor RUNX1 promotes survival of acute myeloid leukemia cells. *J. Clin. Invest.* 123, 3876-3888. 10.1172/JCI68557 23979164 PMC3754260

[DEV149419C30] Growney J. D., Shigematsu H., Li Z., Lee B. H., Adelsperger J., Rowan R., Curley D. P., Kutok J. L., Akashi K., Williams I. R. et al. (2005). Loss of Runx1 perturbs adult hematopoiesis and is associated with a myeloproliferative phenotype. *Blood* 106, 494-504. 10.1182/blood-2004-08-3280 15784726 PMC1895175

[DEV149419C31] Huang G., Shigesada K., Ito K., Wee H.-J., Yokomizo T. and Ito Y. (2001). Dimerization with PEBP2beta protects RUNX1/AML1 from ubiquitin-proteasome-mediated degradation. *EMBO J.* 20, 723-733. 10.1093/emboj/20.4.723 11179217 PMC145428

[DEV149419C32] Ichikawa M., Asai T., Saito T., Yamamoto G., Seo S., Yamazaki I., Yamagata T., Mitani K., Chiba S., Hirai H., et al. (2004). AML-1 is required for megakaryocytic maturation and lymphocytic differentiation, but not for maintenance of hematopoietic stem cells in adult hematopoiesis. *Nat. Med.* 10, 299-304. 10.1038/nm997 14966519

[DEV149419C33] Jaffredo T., Gautier R., Eichmann A. and Dieterlen-Lievre F. (1998). Intraaortic hemopoietic cells are derived from endothelial cells during ontogeny. *Development* 125, 4575-4583. 9778515 10.1242/dev.125.22.4575

[DEV149419C34] Kagoshima H., Akamatsu Y., Ito Y. and Shigesada K. (1996). Functional dissection of the alpha and beta subunits of transcription factor PEBP2 and the redox susceptibility of its DNA binding activity. *J. Biol. Chem.* 271, 33074-33082. 10.1074/jbc.271.51.33074 8955155

[DEV149419C35] Kim S. Y., Theunissen J.-W., Balibalos J., Liao-Chan S., Babcock M. C., Wong T., Cairns B., Gonzalez D., van der Horst E. H., Perez M. et al. (2015). A novel antibody-drug conjugate targeting SAIL for the treatment of hematologic malignancies. *Blood Cancer J.* 5, e316 10.1038/bcj.2015.39 26024286 PMC4476018

[DEV149419C36] Kissa K. and Herbomel P. (2010). Blood stem cells emerge from aortic endothelium by a novel type of cell transition. *Nature* 464, 112-115. 10.1038/nature08761 20154732

[DEV149419C37] Kyba M., Perlingeiro R. C. R. and Daley G. Q. (2002). HoxB4 confers definitive lymphoid-myeloid engraftment potential on embryonic stem cell and yolk sac hematopoietic progenitors. *Cell* 109, 29-37. 10.1016/S0092-8674(02)00680-3 11955444

[DEV149419C38] Lacaud G., Gore L., Kennedy M., Kouskoff V., Kingsley P., Hogan C., Carlsson L., Speck N., Palis J. and Keller G. (2002). Runx1 is essential for hematopoietic commitment at the hemangioblast stage of development in vitro. *Blood* 100, 458-466. 10.1182/blood-2001-12-0321 12091336

[DEV149419C39] Lacaud G., Kouskoff V., Trumble A., Schwantz S. and Keller G. (2004). Haploinsufficiency of Runx1 results in the acceleration of mesodermal development and hemangioblast specification upon in vitro differentiation of ES cells. *Blood* 103, 886-889. 10.1182/blood-2003-06-2149 14525762

[DEV149419C40] Lam E. Y. N., Hall C. J., Crosier P. S., Crosier K. E. and Flores M. V. (2010). Live imaging of Runx1 expression in the dorsal aorta tracks the emergence of blood progenitors from endothelial cells. *Blood* 116, 909-914. 10.1182/blood-2010-01-264382 20453160

[DEV149419C41] Lancrin C., Sroczynska P., Stephenson C., Allen T., Kouskoff V. and Lacaud G. (2009). The haemangioblast generates haematopoietic cells through a haemogenic endothelium stage. *Nature* 457, 892-895. 10.1038/nature07679 19182774 PMC2661201

[DEV149419C42] Lancrin C., Sroczynska P., Serrano A. G., Gandillet A., Ferreras C., Kouskoff V. and Lacaud G. (2010). Blood cell generation from the hemangioblast. *J. Mol. Med. (Berl.)* 88, 167-172. 10.1007/s00109-009-0554-0 19856139

[DEV149419C43] Lancrin C., Mazan M., Stefanska M., Patel R., Lichtinger M., Costa G., Vargel O., Wilson N. K., Moroy T., Bonifer C. et al. (2012). GFI1 and GFI1B control the loss of endothelial identity of hemogenic endothelium during hematopoietic commitment. *Blood* 120, 314-322. 10.1182/blood-2011-10-386094 22668850

[DEV149419C44] Levanon D. and Groner Y. (2004). Structure and regulated expression of mammalian RUNX genes. *Oncogene* 23, 4211-4219. 10.1038/sj.onc.1207670 15156175

[DEV149419C45] Lie-A-Ling M., Marinopoulou E., Li Y., Patel R., Stefanska M., Bonifer C., Miller C., Kouskoff V. and Lacaud G. (2014). RUNX1 positively regulates a cell adhesion and migration program in murine hemogenic endothelium prior to blood emergence. *Blood* 124, e11-e20. 10.1182/blood-2014-04-572958 25082880

[DEV149419C46] Lilly A. J., Costa G., Largeot A., Fadlullah M. Z., Lie-A-Ling M., Lacaud G. and Kouskoff V. (2016a). Interplay between SOX7 and RUNX1 regulates hemogenic endothelial fate in the yolk sac. *Development*. 143, 4341-4351. 10.1242/dev.140970 27802172

[DEV149419C47] Lilly A. J., Lacaud G. and Kouskoff V. (2016b). SOXF transcription factors in cardiovascular development. *Semin. Cell Dev. Biol.* 63, 50-57. 10.1016/j.semcdb.2016.07.021 27470491

[DEV149419C48] Lizama C. O., Hawkins J. S., Schmitt C. E., Bos F. L., Zape J. P., Cautivo K. M., Borges Pinto H., Rhyner A. M., Yu H., Donohoe M. E. et al. (2015). Repression of arterial genes in hemogenic endothelium is sufficient for haematopoietic fate acquisition. *Nat. Commun.* 6, 7739 10.1038/ncomms8739 26204127 PMC4519987

[DEV149419C49] Matheny C. J., Speck M. E., Cushing P. R., Zhou Y., Corpora T., Regan M., Newman M., Roudaia L., Speck C. L., Gu T.-L. et al. (2007). Disease mutations in RUNX1 and RUNX2 create nonfunctional, dominant-negative, or hypomorphic alleles. *EMBO J.* 26, 1163-1175. 10.1038/sj.emboj.7601568 17290219 PMC1852839

[DEV149419C50] Mavilio F., Ferrari G., Rossini S., Nobili N., Bonini C., Casorati G., Traversari C. and Bordignon C. (1994). Peripheral blood lymphocytes as target cells of retroviral vector-mediated gene transfer. *Blood* 83, 1988-1997. 8142665

[DEV149419C51] Miyoshi H., Ohira M., Shimizu K., Mitani K., Hirai H., Imai T., Yokoyama K., Soceda E. and Ohkl M. (1995). Alternative splicing and genomic structure of the AML1 gene involved in acute myeloid leukemia. *Nucleic Acids Res.* 23, 2762-2769. 10.1093/nar/23.14.2762 7651838 PMC307102

[DEV149419C52] Nagata T., Gupta V., Sorce D., Kim W.-Y., Sali A., Chait B. T., Shigesada K., Ito Y. and Werner M. H. (1999). Immunoglobulin motif DNA recognition and heterodimerization of the PEBP2/CBF Runt domain. *Nat. Struct. Biol.* 6, 615-619. 10.1038/10658 10404214

[DEV149419C53] Nieke S., Yasmin N., Kakugawa K., Yokomizo T., Muroi S. and Taniuchi I. (2017). Unique N-terminal sequences in two Runx1 isoforms are dispensable for Runx1 function. *BMC Dev. Biol.* 17, 81 10.1186/s12861-017-0156-y PMC5648507 29047338

[DEV149419C54] Niki M., Okada H., Takano H., Kuno J., Tani K., Hibino H., Asano S., Ito Y., Satake M. and Noda T. (1997). Hematopoiesis in the fetal liver is impaired by targeted mutagenesis of a gene encoding a non-DNA binding subunit of the transcription factor, polyomavirus enhancer binding protein 2/core binding factor. *Proc. Natl. Acad. Sci. USA* 94, 5697-5702. 10.1073/pnas.94.11.5697 9159135 PMC20841

[DEV149419C55] North T., Gu T. L., Stacy T., Wang Q., Howard L., Binder M., Marin-Padilla M. and Speck N. A. (1999). Cbfa2 is required for the formation of intra-aortic hematopoietic clusters. *Development* 126, 2563-2575. 10226014 10.1242/dev.126.11.2563

[DEV149419C56] Ogawa E., Inuzuka M., Maruyama M., Satake M., Naito-Fujimoto M., Ito Y. and Shigesada K. (1993). Molecular cloning and characterization of PEBP2β, the heterodimeric partner of a novel Drosophila runt-related DNA binding protein PEBP2α. *Virology* 194, 314-331. 10.1006/viro.1993.1262 8386878

[DEV149419C57] Okuda T., van Deursen J., Hiebert S. W., Grosveld G. and Downing J. R. (1996). AML1, the target of multiple chromosomal translocations in human leukemia, is essential for normal fetal liver hematopoiesis. *Cell* 84, 321-330. 10.1016/S0092-8674(00)80986-1 8565077

[DEV149419C58] Pozner A., Lotem J., Xiao C., Goldenberg D., Brenner O., Negreanu V., Levanon D. and Groner Y. (2007). Developmentally regulated promoter-switch transcriptionally controls Runx1 function during embryonic hematopoiesis. *BMC Dev. Biol.* 7, 84 10.1186/1471-213X-7-84 17626615 PMC1941738

[DEV149419C59] Ptasinska A., Assi S. A., Martinez-Soria N., Imperato M. R., Piper J., Cauchy P., Pickin A., James S. R., Hoogenkamp M., Williamson D. et al. (2014). Identification of a dynamic core transcriptional network in t(8;21) AML that regulates differentiation block and self-renewal. *Cell Rep.* 8, 1974-1988. 10.1016/j.celrep.2014.08.024 25242324 PMC4487811

[DEV149419C60] Ran F. A., Hsu P. D., Wright J., Agarwala V., Scott D. A. and Zhang F. (2013). Genome engineering using the CRISPR-Cas9 system. *Nat. Protoc.* 8, 2281-2308. 10.1038/nprot.2013.143 24157548 PMC3969860

[DEV149419C61] Rio-Machin A., Menezes J., Maiques-Diaz A., Agirre X., Ferreira B. I., Acquadro F., Rodriguez-Perales S., Juaristi K. A., Alvarez S. and Cigudosa J. C. (2012). Abrogation of RUNX1 gene expression in de novo myelodysplastic syndrome with t(4;21)(q21;q22). *Haematologica* 97, 534-537. 10.3324/haematol.2011.050567 22102704 PMC3347656

[DEV149419C62] Sasaki K., Yagi H., Bronson R. T., Tominaga K., Matsunashi T., Deguchi K., Tani Y., Kishimoto T. and Komori T. (1996). Absence of fetal liver hematopoiesis in mice deficient in transcriptional coactivator core binding factor beta. *Proc. Natl. Acad. Sci. USA* 93, 12359-12363. 10.1073/pnas.93.22.12359 8901586 PMC37996

[DEV149419C63] Seppen J., Barry S. C., Klinkspoor J. H., Katen L. J., Lee S. P., Garcia J. V. and Osborne W. R. A. (2000). Apical gene transfer into quiescent human and canine polarized intestinal epithelial cells by lentivirus vectors. *J. Virol.* 74, 7642-7645. 10.1128/JVI.74.16.7642-7645.2000 10906219 PMC112286

[DEV149419C64] Song W.-J., Sullivan M. G., Legare R. D., Hutchings S., Tan X., Kufrin D., Ratajczak J., Resende I. C., Haworth C., Hock R. et al. (1999). Haploinsufficiency of CBFA2 causes familial thrombocytopenia with propensity to develop acute myelogenous leukaemia. *Nat. Genet.* 23, 166-175. 10.1038/13793 10508512

[DEV149419C65] Sood R., English M. A., Belele C. L., Jin H., Bishop K., Haskins R., McKinney M. C., Chahal J., Weinstein B. M., Wen Z. et al. (2010). Development of multilineage adult hematopoiesis in the zebrafish with a runx1 truncation mutation. *Blood* 115, 2806-2809. 10.1182/blood-2009-08-236729 20154212 PMC2854427

[DEV149419C66] Sroczynska P., Lancrin C., Kouskoff V. and Lacaud G. (2009a). The differential activities of Runx1 promoters define milestones during embryonic hematopoiesis. *Blood* 114, 5279-5289. 10.1182/blood-2009-05-222307 19858498

[DEV149419C67] Sroczynska P., Lancrin C., Pearson S., Kouskoff V. and Lacaud G. (2009b). In vitro differentiation of embryonic stem cells as a model of early hematopoietic development. *Methods Mol. Biol.* 538, 317-334. 10.1007/978-1-59745-418-6_16 19277585

[DEV149419C68] Swiers G., Baumann C., O'Rourke J., Giannoulatou E., Taylor S., Joshi A., Moignard V., Pina C., Bee T., Kokkaliaris K. D. et al. (2013). Early dynamic fate changes in haemogenic endothelium characterized at the single-cell level. *Nat. Commun.* 4, 2924 10.1038/ncomms3924 24326267 PMC4362711

[DEV149419C69] Tang Y.-Y., Crute B. E., Kelley J. J., Huang X., Yan J., Shi J., Hartman K. L., Laue T. M., Speck N. A. and Bushweller J. H. (2000). Biophysical characterization of interactions between the core binding factor alpha and beta subunits and DNA. *FEBS Lett.* 470, 167-172. 10.1016/S0014-5793(00)01312-0 10734228

[DEV149419C70] Telfer J. C. and Rothenberg E. V. (2001). Expression and function of a stem cell promoter for the murine CBFalpha2 gene: distinct roles and regulation in natural killer and T cell development. *Dev. Biol.* 229, 363-382. 10.1006/dbio.2000.9991 11203699

[DEV149419C71] Thambyrajah R., Mazan M., Patel R., Moignard V., Stefanska M., Marinopoulou E., Li Y., Lancrin C., Clapes T., Möröy T. et al. (2016). GFI1 proteins orchestrate the emergence of haematopoietic stem cells through recruitment of LSD1. *Nat. Cell Biol.* 18, 21-32. 10.1038/ncb3276 26619147

[DEV149419C72] Wang Q., Stacy T., Binder M., Marin-Padilla M., Sharpe A. H. and Speck N. A. (1996a). Disruption of the Cbfa2 gene causes necrosis and hemorrhaging in the central nervous system and blocks definitive hematopoiesis. *Proc. Natl. Acad. Sci. USA* 93, 3444-3449. 10.1073/pnas.93.8.3444 8622955 PMC39628

[DEV149419C73] Wang Q., Stacy T., Miller J. D., Lewis A. F., Gu T.-L., Huang X., Bushweller J. H., Bories J.-C., Alt F. W., Ryan G. et al. (1996b). The CBFbeta subunit is essential for CBFalpha2 (AML1) function in vivo. *Cell* 87, 697-708. 10.1016/S0092-8674(00)81389-6 8929538

[DEV149419C74] Yanagida M., Osato M., Yamashita N., Liqun H., Jacob B., Wu F., Cao X., Nakamura T., Yokomizo T., Takahashi S. et al. (2005). Increased dosage of Runx1/AML1 acts as a positive modulator of myeloid leukemogenesis in BXH2 mice. *Oncogene* 24, 4477-4485. 10.1038/sj.onc.1208675 15856017

[DEV149419C75] Yokomizo T., Yanagida M., Huang G., Osato M., Honda C., Ema M., Takahashi S., Yamamoto M. and Ito Y. (2008). Genetic evidence of PEBP2β-independent activation of Runx1 in the murine embryo. *Int. J. Hematol.* 88, 134-138. 10.1007/s12185-008-0121-4 18594778

[DEV149419C76] Yoon D., Kim B. and Prchal J. T. (2008). Cre recombinase expression controlled by the hematopoietic regulatory domain of Gata-1 is erythroid-specific. *Blood Cells Mol. Dis.* 40, 381-387. 10.1016/j.bcmd.2007.10.008 18077194

[DEV149419C77] Zufferey R., Dull T., Mandel R. J., Bukovsky A., Quiroz D., Naldini L. and Trono D. (1998). Self-inactivating lentivirus vector for safe and efficient in vivo gene delivery. *J. Virol.* 72, 9873-9880. 9811723 10.1128/jvi.72.12.9873-9880.1998 PMC110499

