# Supplementary material for: Regulation of RUNX1 dosage is crucial for efficient blood formation from hemogenic endothelium
Source: Development. 2018 Mar 1;145(5):dev149419. doi: 10.1242/dev.149419 (PMC5868988; doi:10.1242/dev.149419)
Supplement: Supplementary information [file develop-145-149419-s1.pdf]

Supplemental Materials and Methods

Runx1b Lentiviral vectors

All lentiviral transfer vector used in this study have the same backbone as EF1-eGFP described previously (Gilham et al., 2010; Seppen et al., 2000). VSVg pseudotyped lentiviral vectors were produced using the third-generation self-inactivating lentiviral vector system as described previously (Dull et al., 1998; Seppen et al., 2000; Zufferey et al., 1998). Briefly, viral supernatant was harvested from transfected HEK293T cells 2, 3 and 4 days post-transfection. Supernatant was passed through a 0.45 µm filter (Millipore, Billerica, MA, USA), concentrated by ultra-centrifugation (2h at 20000 r.p.m., 47000k, 4 ◦C; Optima L-90K ultracentrifuge; Beckman Coulter, Fullerton, CA, USA), resuspended in phosphate-buffered saline, aliquoted and stored at −80◦C. The murine proximal *Runx1* isoform (*Runx1b*) cDNA (Telfer and Rothenberg, 2001) inserted in the iRunx1ko mESC was PCR amplified and used to construct all lentiviral vectors. Point mutations were introduced with the QuikChange II Site-Directed Mutagenesis Kit according to manufacturers's protocol (Agilent Technologies). All mutations have been characterized previously (Matheny et al., 2007). The truncated construct was generated by inserting a stop codon at the required aa by PCR. All constructs were verified by sequencing and western blotting. Primers: Table S2. Transductions of FLK1+ cells was performed as previously described (Gandillet et al., 2009).

Inducible *Sox7* PiggyBAC transposon

Doxycycline inducible PiggyBAC transposon plasmid PB-TRE3G and a PiggyBAC transposase plasmid were kindly provided by Dr Pentao Liu (Wellcome Trust Sanger Institute). The mouse *Sox7* cDNA (Gandillet et al., 2009) was cloned behind the tetracycline responsive element (TRE3G) in PB-TRE3G followed by a SV40 polyadenylation signal. A second cassette was inserted containing the ubiquitously active EF1 promoter (Gilham et al., 2010), truncated human nerve growth factor receptor cDNA (th*NGFR*) (Mavilio et al., 1994) and a bovine growth hormone polyadenylation signal (PB-TRE3G_Sox7). Mouse ESC containing PB-TRE3G_Sox7 were established by co-transfection with the PiggyBAC transposase using PEI (Bartman et al., 2015). Cell lines were established by magnetic-activated cell sorting (Miltenyi Biotec) for thNGFR (Table S1).

Bartman, C. M., Egelston, J., Ren, X., Das, R. and Phiel, C. J. (2015). A simple and efficient method for transfecting mouse embryonic stem cells using polyethylenimine. *Exp Cell Res* **330**, 178-185.
Dull, T., Zufferey, R., Kelly, M., Mandel, R. J., Nguyen, M., Trono, D. and Naldini, L. (1998). A third-generation lentivirus vector with a conditional packaging system. *J Virol* **72**, 8463-8471.
Gandillet, A., Serrano, A. G., Pearson, S., Lie-A-Ling, M., Lacaud, G. and Kouskoff, V. (2009). Sox7-sustained expression alters the balance between proliferation and differentiation of hematopoietic progenitors at the onset of blood specification. *Blood* **114**, 4813-4822.
Gilham, D. E., Lie-A-Ling, M., Taylor, N. and Hawkins, R. E. (2010). Cytokine stimulation and the choice of promoter are critical factors for the efficient transduction of mouse T cells with HIV-1 vectors. *J Gene Med* **12**, 129-136.
Matheny, C. J., Speck, M. E., Cushing, P. R., Zhou, Y., Corpora, T., Regan, M., Newman, M., Roudaia, L., Speck, C. L., Gu, T. L., et al. (2007). Disease mutations in RUNX1 and RUNX2 create nonfunctional, dominant-negative, or hypomorphic alleles. *The EMBO journal* **26**, 1163-1175.
Mavilio, F., Ferrari, G., Rossini, S., Nobili, N., Bonini, C., Casorati, G., Traversari, C. and Bordignon, C. (1994). Peripheral blood lymphocytes as target cells of retroviral vector-mediated gene transfer. *Blood* **83**, 1988-1997.
Seppen, J., Barry, S. C., Klinkspoor, J. H., Katen, L. J., Lee, S. P., Garcia, J. V. and Osborne, W. R. (2000). Apical gene transfer into quiescent human and canine polarized intestinal epithelial cells by lentivirus vectors. *J Virol* **74**, 7642-7645.
Telfer, J. C. and Rothenberg, E. V. (2001). Expression and function of a stem cell promoter for the murine CBFalpha2 gene: distinct roles and regulation in natural killer and T cell development. *Dev Biol* **229**, 363-382.
Zufferey, R., Dull, T., Mandel, R. J., Bukovsky, A., Quiroz, D., Naldini, L. and Trono, D. (1998). Self-inactivating lentivirus vector for safe and efficient in vivo gene delivery. *J Virol* **72**, 9873-9880.

Supplementary table S1: Antibodies

Target	antibody	clone	manufacturer	catalog #	application	dilution
FLK1 / CD309 / VEGFR2 / KDR	Anti-CD309 -BIOTIN	Avas12	Miltenyi Biotec	130101915	MACS	1:100
CD271 / thNGF	Anti-CD271-BIOTIN	C40-1457	BD Pharmingen	557195	MACS	1:100
CD41 / ITGA2B	Anti-CD41 -FITC	MWReg30	eBioscience	11-0411	FACS	1:120
CD41 / ITGA2B	Anti-CD41 -PE-CY7	MWReg30	eBioscience	25-0411	FACS	1:120
TIE2 / TEK / CD202	Anti-Tie-2-PE	TEK4	eBioscience	12-5987	FACS	1:300
c-KIT / CD117	Anti-CD117-APC	2B8	eBioscience	17-1171	FACS	1:120
CD34	Anti-CD34-FITC	RAM34	eBioscience	11-0341	FACS	1:200
FcεRIα	Anti-FcεRIα-PE	MAR-1	eBioscience	13-5898	FACS	1:200
CD45 / LCA / Ly-5	Anti-CD45 -PE-CY7	30-F11	eBioscience	25-0451	FACS	1:120
CD45 / LCA / Ly-5	Anti-CD45 -FITC	30-F11	eBioscience	11-0451	FACS	1:120
β-Actin	Anti-β-Actin	8H10D10	Cell Signalling Technology	3700	Western Blot	1:5000
CBFβ / PEBP2β	Anti-PEBP2β	141,4,1	Santa Cruz	sc-56751	Western Blot / Duolink	1:100 / 1:50
RUNX1 / AML1 + RUNX3 + RUNX2	Anti-RUNX	EPR3099	Abcam	ab92336	Western Blot / Duolink	1:5000 / 1:200
SOX7	Anti-SOX7		R&D systems	AF2766	Duolink	1:200

Development • Supplementary information

Supplementary table S2: Oligos

primer name	primer sequence (5'-3')	Universal probe #	target	application
AI467606_F	gcgaaatccaagattgaagc	56	expressed sequence AI467606 (SAIL)	realtime pcr
AI467606_R	aacagtcaggaacgtctccact	56	expressed sequence AI467606 (SAIL)	realtime pcr
B-Actin_F	tgacaggatgcagaaggaga	106	actin, beta	realtime pcr
B-Actin_R	cgctcaggaggagcaatg	106	actin, beta	realtime pcr
Cbfb_F	tatgggttgcctggagtttg	3	Core binding factor beta	realtime pcr
Cbfb_R	aaggcctgttgtgctaatgc	3	Core binding factor beta	realtime pcr
Cdh5_F	tcatcaaacccacgaagtcc	42	Cadherin 5	realtime pcr
Cdh5_R	ggtctgtggcctcaatgtaga	42	Cadherin 5	realtime pcr
Gfi1_F	atgtgcggcaagaccttc	1	Growth factor independent 1	realtime pcr
Gfi1_R	acagtcaaagctgcgttcct	1	Growth factor independent 1	realtime pcr
Gfi1b_F	agcacagagtctcccttgga	80	Growth factor independent 1B	realtime pcr
Gfi1b_R	atgaggggtggagaacacc	80	Growth factor independent 1B	realtime pcr
Itgb3_F	tgaccggaaggaatttgcta	21	Integrin beta 3 (CD61)	realtime pcr
Itgb3_R	acagcgggttgtttgctg	21	Integrin beta 3 (CD61)	realtime pcr
Sox7_F	cagcaagatgctgggaaag	97	SRY (sex determining region Y)-box 7	realtime pcr
Sox7_F	tgcatcatccacatagggtct	97	SRY (sex determining region Y)-box 7	realtime pcr
Sox17_F	cacaacgcagagctaagcaa	97	SRY-box containing gene 17	realtime pcr
Sox17_R	cgcttctctgccaaggtc	97	SRY-box containing gene 17	realtime pcr
Runx1_Runt_F	ctccgtgctacccactcact	77	Runt related transcription factor 1, Runt domain	realtime pcr
Runx1_Runt_R	atgacggtgaccagagtgc	77	Runt related transcription factor 1, Runt domain	realtime pcr
Runx1Ex8_F	cggttcctaccagttctcca	75	Runt related transcription factor 1, Exon 8	realtime pcr
Runx1Ex8_R	tctccaccacgtcgctct	75	Runt related transcription factor 1, Exon 8	realtime pcr
Runx1_Proximal_F	aagatccgagcccctgtc	17	Runt related transcription factor 1, Proximal isoform	realtime pcr
Runx1_Proximal_R	tcacaacaagccgattgagt	17	Runt related transcription factor 1, Proximal isoform	realtime pcr
Runx1_Distal_F	gaagtgtaagcccagcacagt	40	Runt related transcription factor 1, Distal isoform	realtime pcr
Runx1_Distal_R	ggcgggggattctataattt	40	Runt related transcription factor 1, Distal isoform	realtime pcr
Cbfb_ex4_1F	CACCGGcacactccattcagaatcat	-	mouse Cbfb exon 4	Cbfb exon 4 CRISPR
Cbfb_ex4_1R	AAACatgattctgaatggagtgtgCC	-	mouse Cbfb exon 4	Cbfb exon 4 CRISPR
Cbfb_ex4_2F	CACCGGtctccacagattggatggta	-	mouse Cbfb exon 4	Cbfb exon 4 CRISPR
Cbfb_ex4_2F	AAACtaccatccaatctgtggagaCC	-	mouse Cbfb exon 4	Cbfb exon 4 CRISPR
Runx1 F	gagaagatctGCCACCATGGATTACAAGGATGACGACGATAAGcgtatccccgtagatg	-	mouse Runx1b forward	Runx1b lentivirus
Runx1 R	gagactcgagTCAgtagggccgccacacgg	-	mouse Runx1b reverse	Runx1b lentivirus
Runx1 aa del242 R	gagactcgagTCActgcatctgactttgag	-	mouse Runx1b aa242 truncation	Runx1b lentivirus
Runx1_R139Q_F	aggtttgtcgggcagagcggtagaggc	-	mouse Runx1b mutagenesis R_139_Q mutant	Runx1b lentivirus
Runx1_R139Q _R	gcctctaccgctctgcccgacaaacct	-	mouse Runx1b mutagenesis R_139_Q mutant	Runx1b lentivirus
Runx1_R174Q_F	tggacggcccccaagaaccccgaag	-	mouse Runx1b mutagenesis R_174_Q mutant	Runx1b lentivirus
Runx1 R174Q _R	cttcggggttcttgggggccgtcca	-	mouse Runx1b mutagenesis R_174_Q mutant	Runx1b lentivirus
Runx1 L148F_T149AF	gcaagagcttcactttcgccatcaccgtctttaca	-	mouse Runx1b mutagenesis L148F + T149A mutant	Runx1b lentivirus
Runx1 L148F_T149AR	tgtaaagacggtgatggcgaaagtgaagctcttgc	-	mouse Runx1b mutagenesis L148F + T149A mutant	Runx1b lentivirus
Runx1 T161A_F	cgccacaagttgccgcctaccatagagcc	-	mouse Runx1b mutagenesis T161A mutant	Runx1b lentivirus
Runx1 T161A_R	ggctctatggtaggcggcaacttgtggcg	-	mouse Runx1b mutagenesis T161A mutant	Runx1b lentivirus

Development • Supplementary information

Supplementary table S3: Flowcytometry analyzers

Analyser	Manufacturer
BD FACSCalibur	Becton Dickinson Biosciences
BD FACSCAnto II	Becton Dickinson Biosciences
BD LSR II	Becton Dickinson Biosciences
Novocyte	ACEA Biosciences

Development • Supplementary information

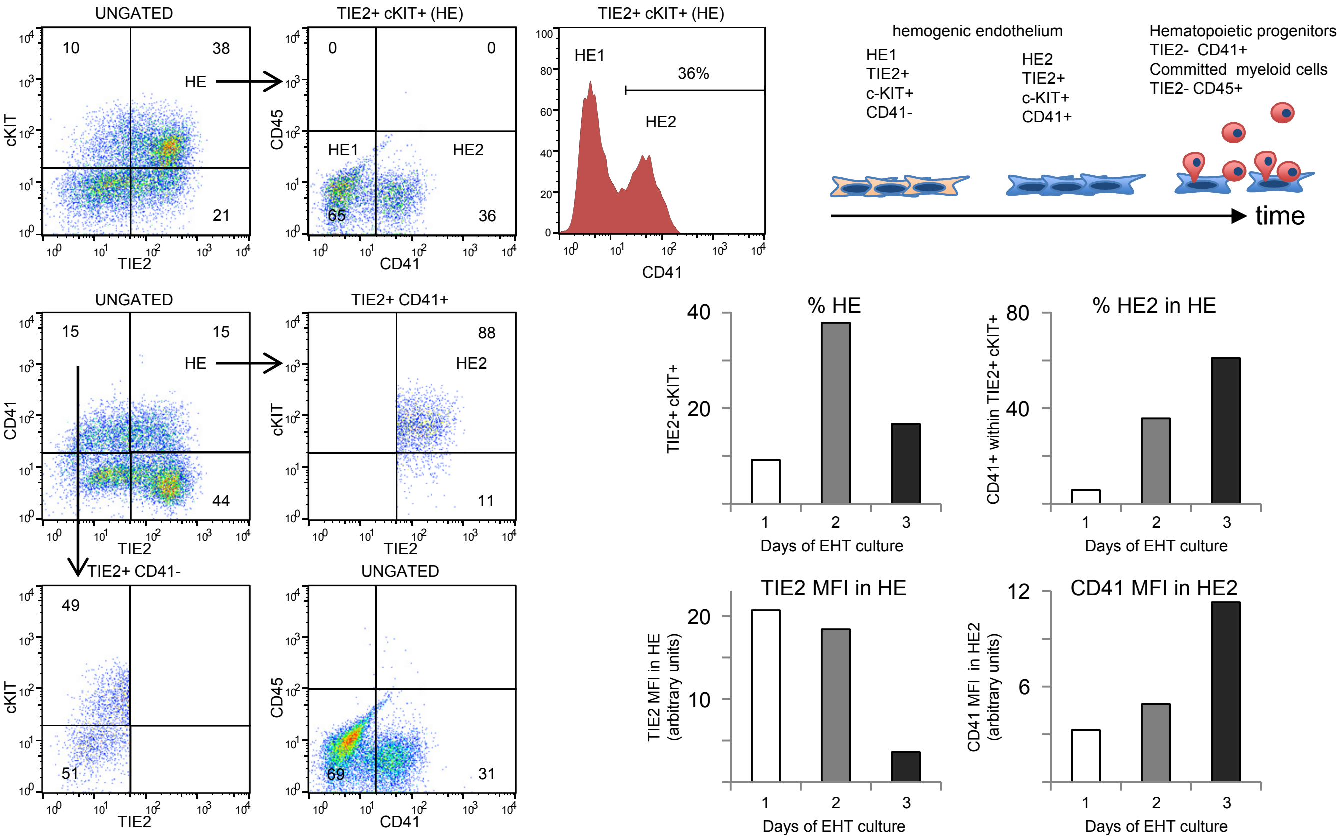

Supplemental figure 1. Hemogenic endothelium in mESC derived EHT-cultures

Left: Flowcytometry plots depicting the hemogenic endothelium 1 (HE1) and hemogenic endothelium 2 (HE2) populations in day two wildtype EHT-cultures. Top right: Schematic of hemogenic endothelium differentiation in mESC derived EHT-cultures. TIE2+/c-KIT+/CD41- HE1 gives rise to Tie2+/c-KIT+/CD41+ HE2 followed by TIE2-/CD41+ hematopoietic progenitors and TIE2-/CD45+ committed myeloid cells. Bottom right: example flow cytometry data for day one to three of wildtype EHT-cultures.

Development • Supplementary information

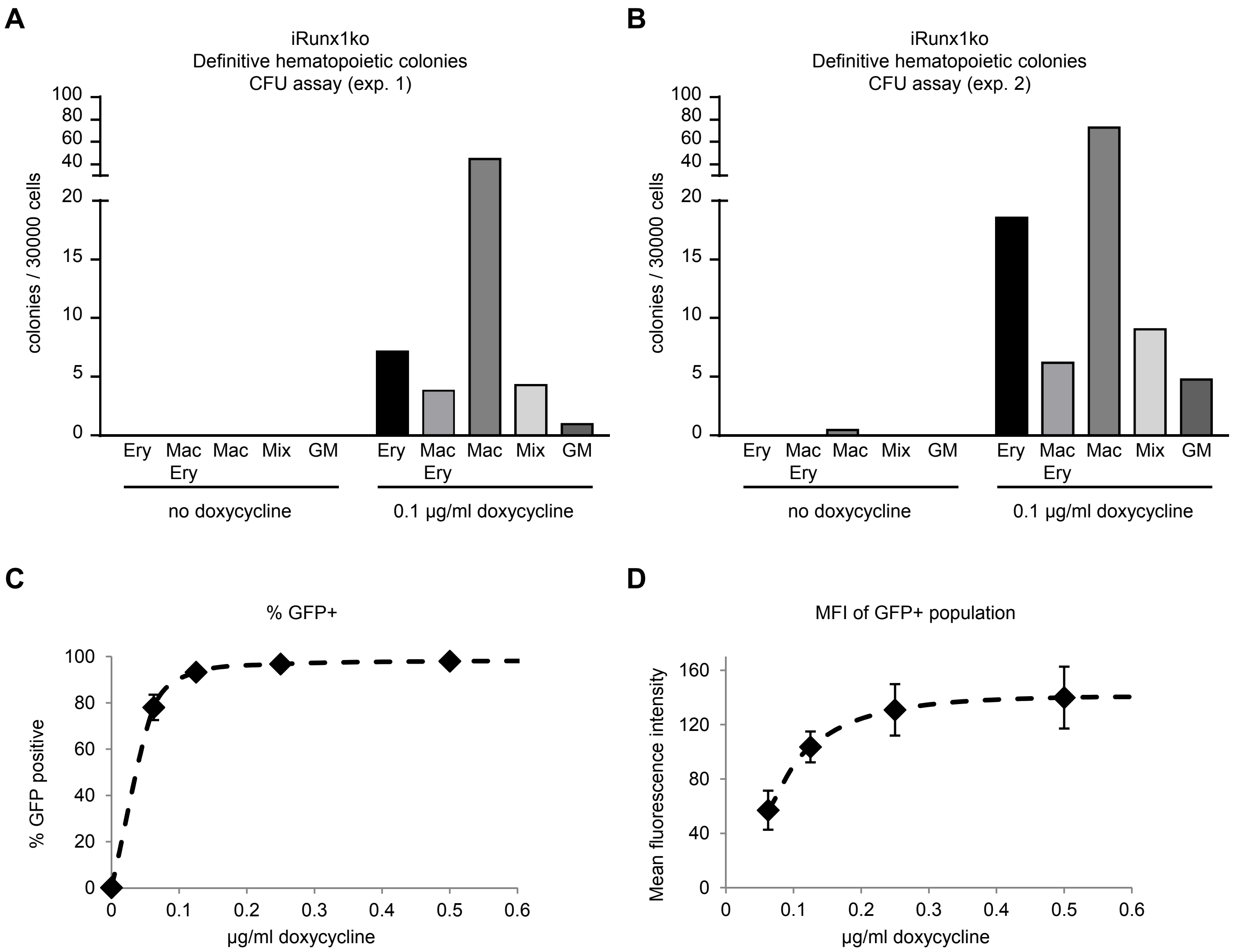

Supplemental figure 2. Rescue of definitive hematopoiesis in iRunx1ko and doxycycline titration on mESC. (A) & (B) Two independent definitive hematopoietic colony formation assays with iRunx1ko replated after four days of EHT-culture. Doxycycline was added at day two of EHT-culture and absent during the CFU-assay. Ery= definitive erythrocyte, Mac/Ery = mixed macrophage erythrocyte, Mac= macrophage, Mix= contains cells of multiple lineages, GM= granulocyte monocyte. Mean of N=3 technical replicates is shown. **(C) & (D)** Doxycycline titration on three independently established mESC lines in which Green Fluorescent Protein (GFP) was under the control of the doxycycline inducible tet-on system. Cells were analyzed by flowcytometry 48 hours after induction. Doxycycline concentrations: 0.0625, 0.125, 0.25, 0.5, 1.0 µg/ml **(C)** percentage of GFP positive cells. Mean±s.d. Results from three clones each analyzed once across three independent experiments are shown. **(D)** Mean fluorescence intensity of the GFP positive populations. Mean±s.d. Results from 3 clones each analyzed once across three independent experiments are shown.

Development • Supplementary information

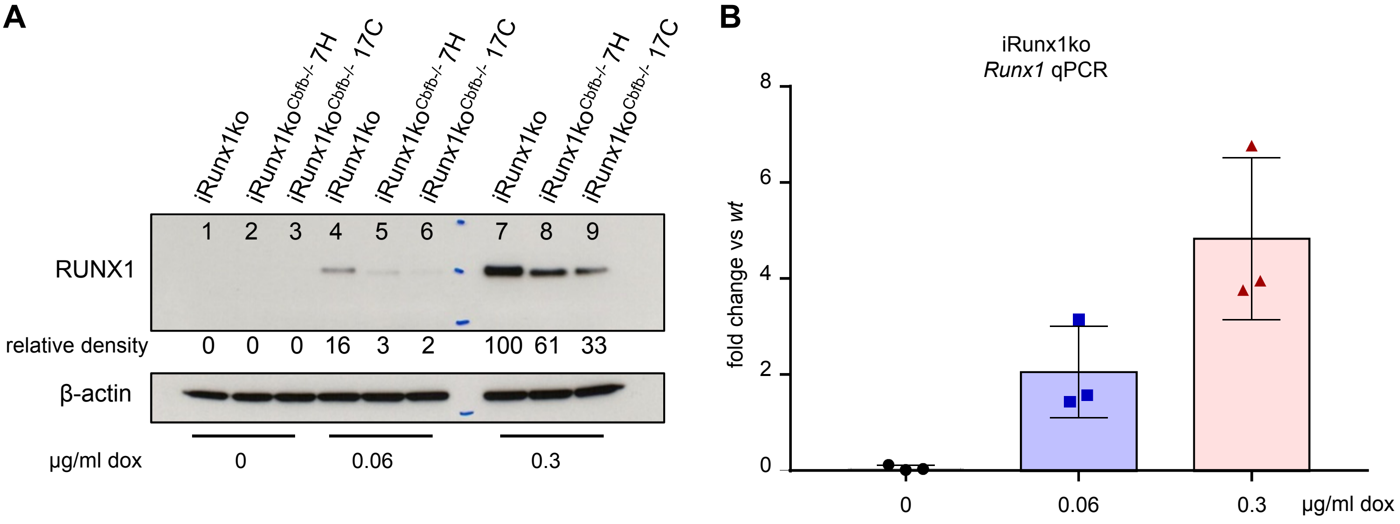

Supplemental figure 3. RUNX1 and CBFβ western blot and qPCR analyses. (A) Western blot of RUNX1 and β-actin in day two EHT-cultures of two iRunx1ko$^{Cbfb-/-}$ clones (lanes 2, 5, 8 and 3, 6, 9) and the parent iRunx1ko line (lane 1, 4, 7) exposed to 0, 0.06 or 0.3 µg/ml doxycycline. β-actin normalized relative density for RUNX1 is shown below the lanes. **(B)** Quantitative PCR analysis of *Runx1* expression in day three iRunx1ko EHT-cultures treated with 0, 0.06 or 0.30 µg/ml doxycycline. Individual biological experiments and Mean±s.d. of N=3 independent differentiations is shown.

Mast-assays

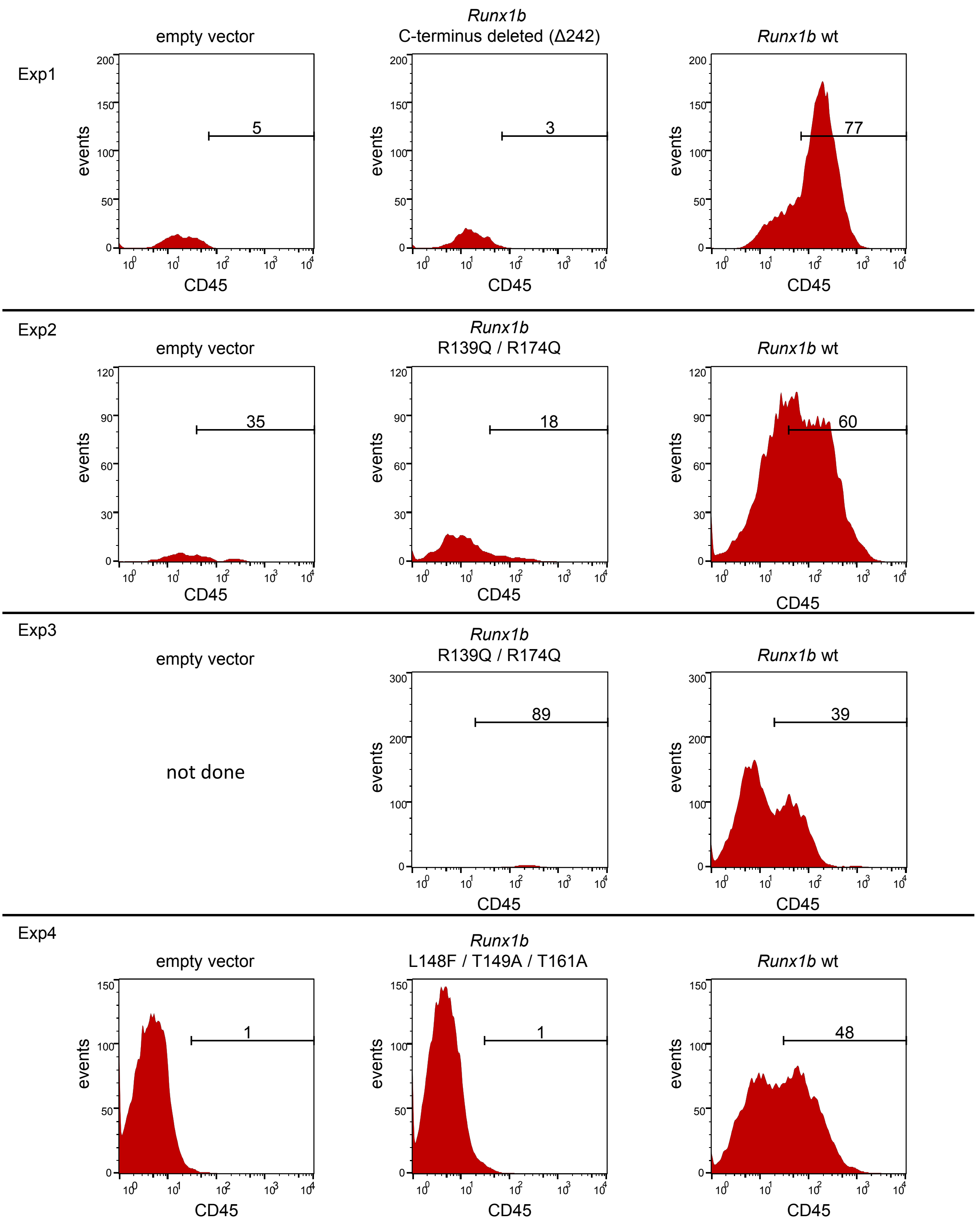

Development • Supplementary information

Supplemental figure 4. Expression of wildtype RUNX1 is essential for the generation of CD45⁺ cells in *Runx1⁻/⁻* mast assays.

Flowcytometry for CD45+ cells for four independent mast cell assays. FLK1+ cells were isolated from *Runx1*$^{-/-}$ mESC differentiated as Embryoid Bodies (EB). The FLK1+ cells were transduced with lentiviral vectors containing either no insert (empty vector), a mutated / truncated *Runx1b* or wildtype *Runx1b* cDNA. Mast cell assays were performed as described in figure 3D. Results show that only cells transduced with the wt *Runx1b* lentivirus can generate CD45+ cells. Neither the empty vector nor the vectors containing truncated *Runx1b* (experiment 1) or mutated *Runx1b* (experiment 2-4) can rescue the formation of CD45^{+} cells from Runx1$^{-/-}$ cells. *Runx1b*Δ242 lacks the C-terminus of RUNX1 which contains the transactivation domain. *Runx1b*_R139Q_R174Q contains two point mutations in the Runt domain which severely impair the DNA binding capacity of RUNX1. *Runx1b*_L148F_T149A_T161A contains three point mutations in the Runt domain which impair both the interaction of RUNX1 with CBFβ and the DNA binding capacity of RUNX1. All mutations have been previously described and characterized by Matheny et al., 2007.

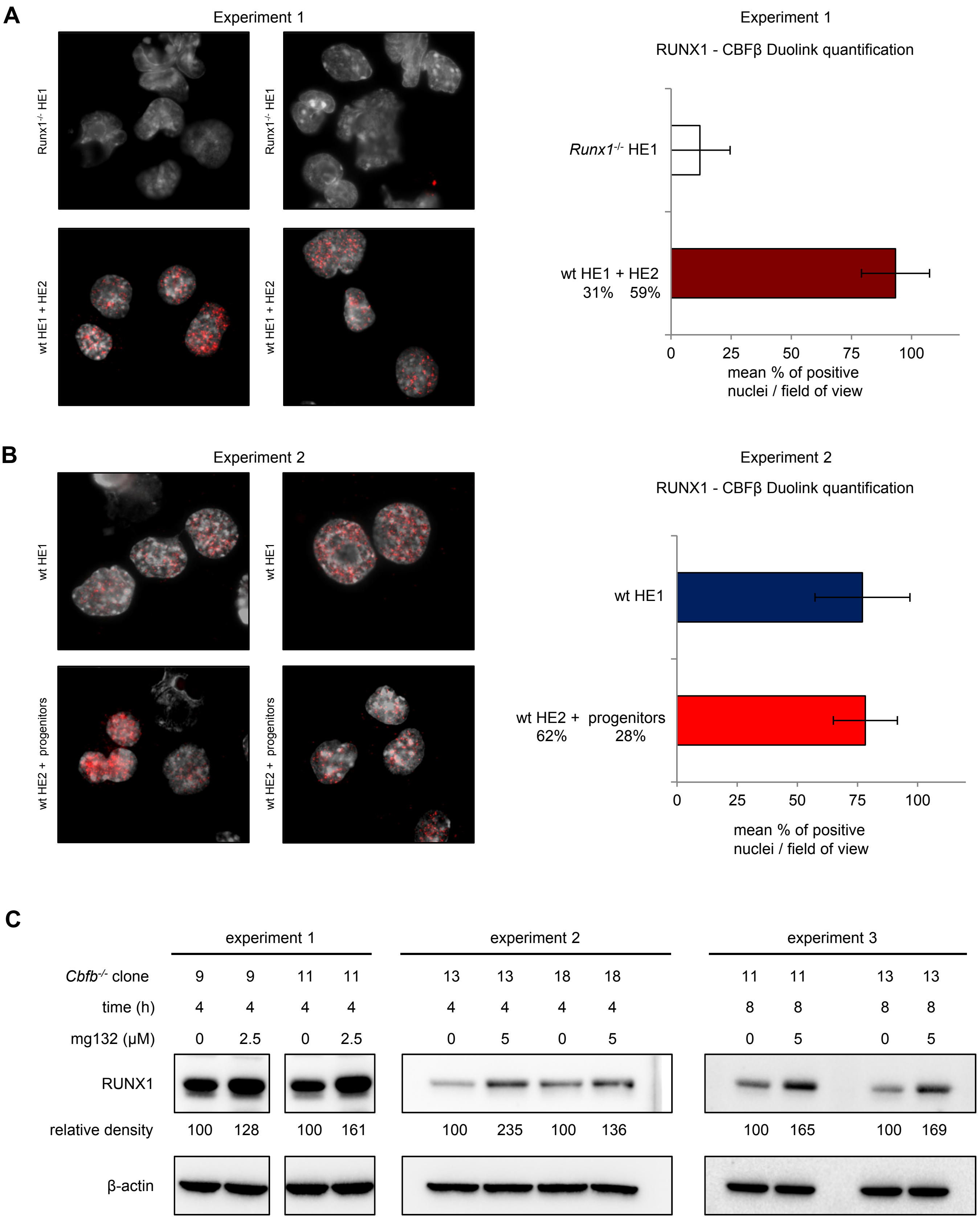
A
Experiment 1
Runx1-/- HE1
Runx1-/- HE1
wt HE1 + HE2
wt HE1 + HE2
Experiment 1
RUNX1 - CBFβ Duolink quantification
Runx1-/- HE1
wt HE1 + HE2
31% 59%
0 25 50 75 100
mean % of positive nuclei / field of view
B
Experiment 2
wt HE1
wt HE1
wt HE2 + progenitors
wt HE2 + progenitors
Experiment 2
RUNX1 - CBFβ Duolink quantification
wt HE1
wt HE2 + progenitors
62% 28%
0 25 50 75 100
mean % of positive nuclei / field of view
C
experiment 1
experiment 2
experiment 3
Cbfb-/- clone 9 9 11 11 13 13 18 18 11 11 13 13
time (h) 4 4 4 4 4 4 4 4 8 8 8 8
mg132 (μM) 0 2.5 0 2.5 0 5 0 5 0 5 0 5
RUNX1
relative density 100 128 100 161 100 235 100 136 100 165 100 169
β-actin

Supplemental figure 5. In mESC derived EHT cultures RUNX1 interacts with CBFβ in the nuclei of the cells and RUNX1 degradation can be reduced by inhibiting the proteasome.

(A and B) Duolink assays for RUNX1 and CBFβ in hemogenic endothelium cells. Each puncta (red) represents a single RUNX1-CBFβ interaction (100x magnification). Grey: DAPI. (A) Duolink on hemogenic endothelium derived from *Runx1*$^{-/-}$ and *Runx1* wt mESC. The proportion of HE1 (Tie2+/c-KIT+/CD41-) and HE2 (TIE2+/c-KIT+/CD41+) as determined by flowcytometry was 31% HE1 and 59% HE2. Graph depicts the mean percentage of positive nuclei / field of view. *Runx1*$^{-/-}$ HE: 6 fields of view (54 nuclei), wildtype HE: 9 fields of view (63 nuclei). (B) Duolink on hemogenic endothelium 1 (TIE2+/c-KIT+/CD41-) and CD41+ cells (62% HE2, 28% progenitors as determined by flow cytometry for TIE2, c-KIT and CD41) derived from *Runx1* wildtype mESC. Graph depicts the mean percentage of positive nuclei / field of view. wildtype HE1: 13 fields of view (129 nuclei) and wildtype CD41+: 13 fields of view (155 nuclei). (C) Western blots for RUNX1 on day three *Cbfb*$^{-/-}$ EHT-cultures treated with 2.5 and 5 μM proteasome inhibitor MG132 for four hours (experiment 1 and 2) or 5 μM for 8 hours (experiment 3). Controls were treated with vehicle (DMSO). β-actin normalized relative density for RUNX1 is shown below the lanes.

Development • Supplementary information

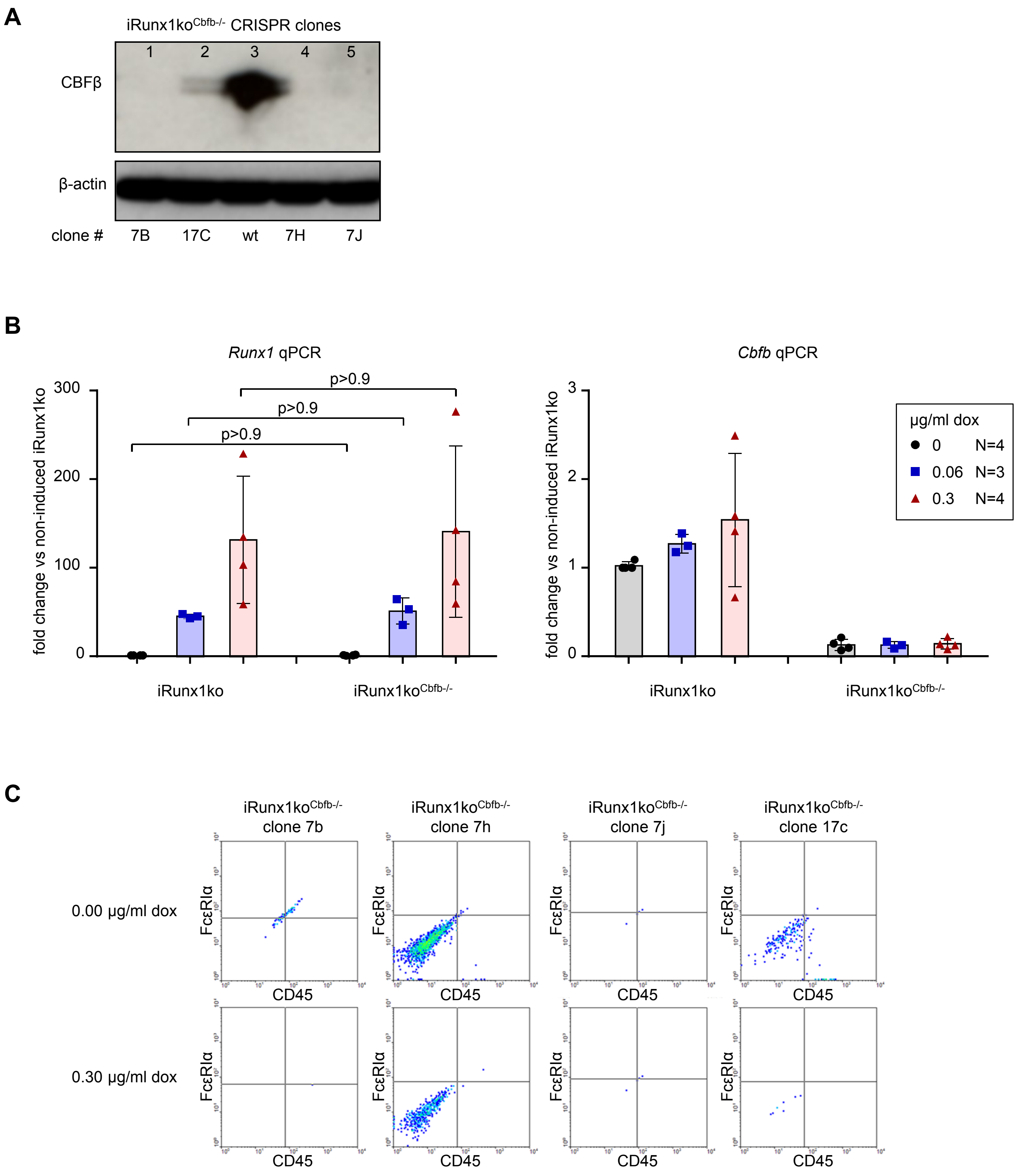

Development • Supplementary information

Supplemental figure 6: iRunx1ko$^{cbfb-/-}$ western blot, qPCR, mast cell assay.

(A) Western blot for CBFβ on CRISPR/Cas9 generated iRunx1ko$^{Cbfb-/-}$ mESC clones (clone 7B, 17C, 7H and 7J) used in this study. Lane 3 contains a *Cbfb* wildtype (wt) mESC sample as a positive control. **(B)** Quantitative PCR on day three EHT-cultures for *Runx1* (left) and *Cbfb* (right) in iRunx1ko$^{Cbfb-/-}$ clones and the parent iRunx1ko line induced with 0, 0.06 or 0.3 µg/ml doxycycline. Data are normalized against the non-induced iRunx1ko line. Each of our four iRunx1$^{Cbfb-/-}$ clone was analysed at least once. Individual biological experiments and Mean$\pm$s.d are shown. Two-way ANOVA. **(C)** Flowcytometry analysis of four independent mast cell differentiations. Each iRunx1ko$^{Cbfb-/-}$ clone was differentiated once. None of the iRunx1ko$^{cbfb-/-}$ clones could generate mast cells either in the presence or absence of doxycycline. The cultures contained very few, if any, non-adherent cells at day 22. These experiments were performed identical to and in parallel with the experiments shown in main figure 3E.

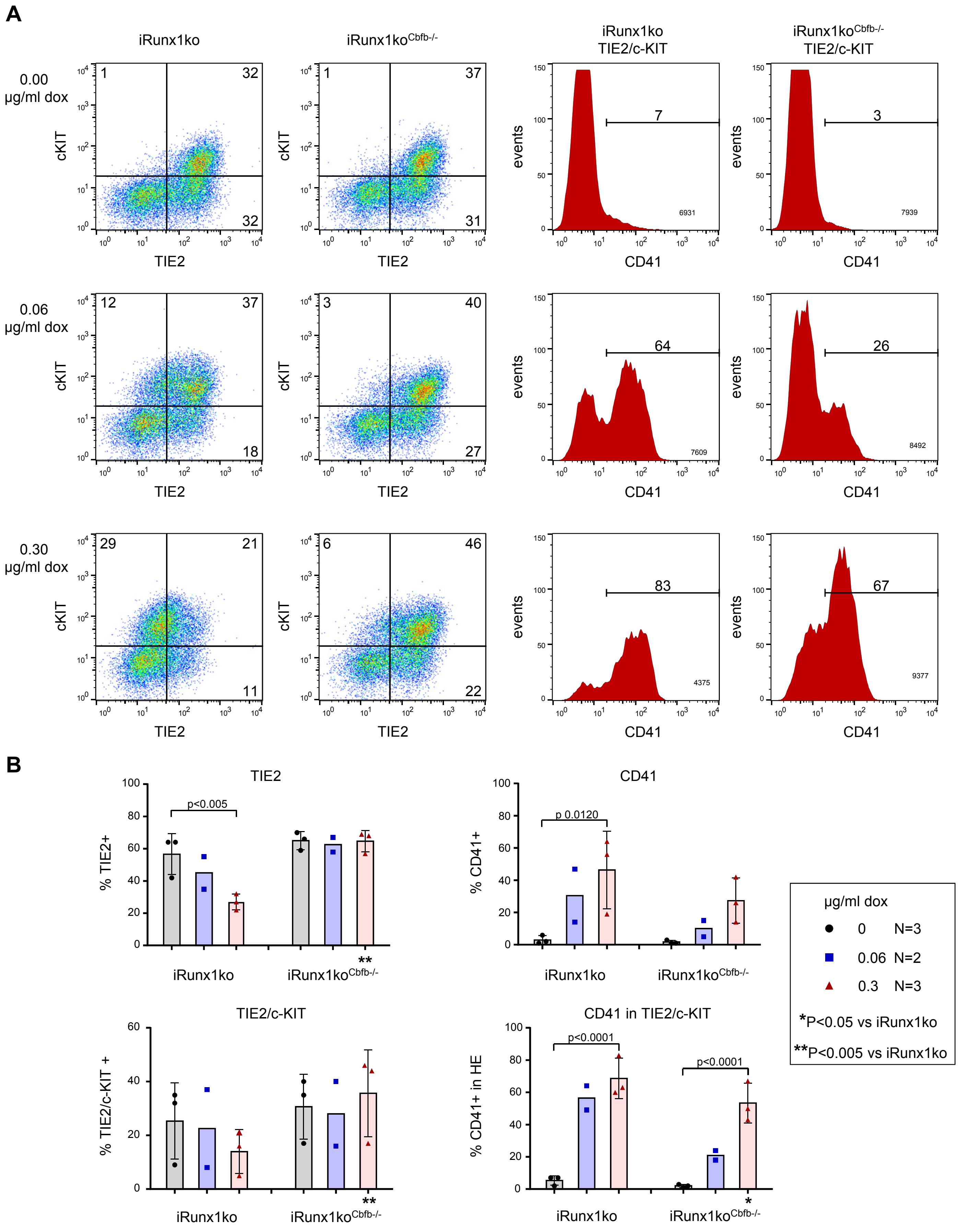
A
iRunx1ko
iRunx1ko Cbfb-/-
iRunx1ko TIE2/c-KIT
iRunx1ko Cbfb-/- TIE2/c-KIT
0.00 µg/ml dox
0.06 µg/ml dox
0.30 µg/ml dox
cKIT
TIE2
events
CD41
B
TIE2
% TIE2+
p<0.005
CD41
% CD41+
p 0.0120
TIE2/c-KIT
% TIE2/c-KIT +
CD41 in TIE2/c-KIT
% CD41+ in HE
p<0.0001
µg/ml dox
0 N=3
0.06 N=2
0.3 N=3
*P<0.05 vs iRunx1ko
**P<0.005 vs iRunx1ko

Supplemental figure 7. Low RUNX1 levels can induce EHT like changes. (A, B) Representative flowcytometry plots **(A)** and flowcytometry data **(B)** of day two iRunx1ko and iRunx1ko$^{Cbfb-/-}$ EHT-cultures. **(A)** Left: plots depicting HE (TIE2+/c-KIT+). Right: TIE2+/c-KIT+ gated histograms depicting the percentage CD41+ cells within the HE. **(B)** Individual biological experiments and Mean±s.d. (only for N>2) are shown. Two-way ANOVA (only for N>2).

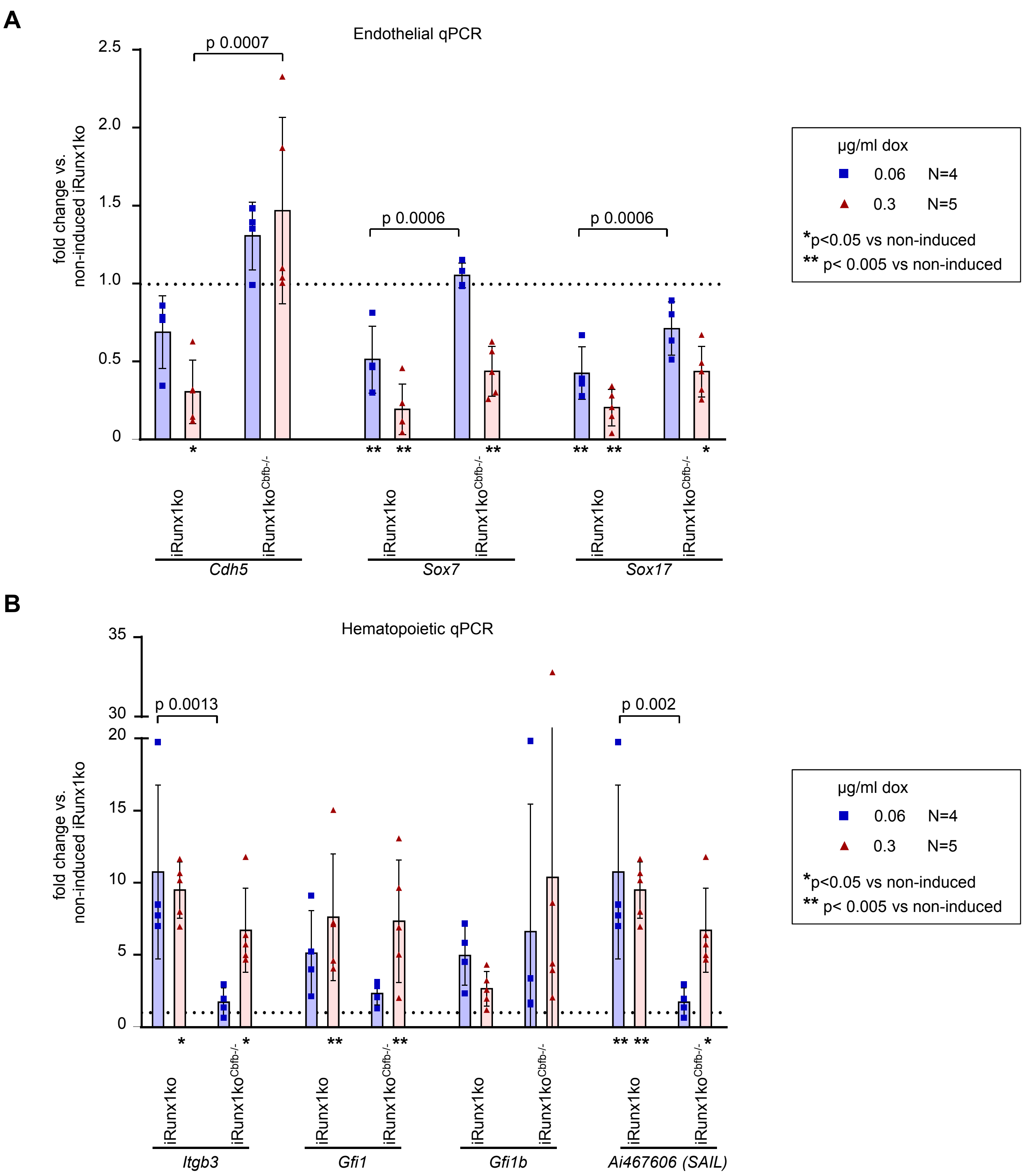

Supplemental figure 8. Quantitative PCR analysis of iRunx1ko and iRunx1ko$^{Cbfb-/-}$ day three EHT culture. qPCR analysis of day three EHT-cultures for endothelial (*Sox7*, *Sox17*, *Cdh5*) **(A)** and hematopoietic genes (*Itgb3*, *Gfi1*, *Gfi1b*, *AI467606*) **(B)**. Data are normalized against the non-induced sample. Individual biological experiments and Mean±s.d. are shown. Two-way ANOVA. N=biological replicates.

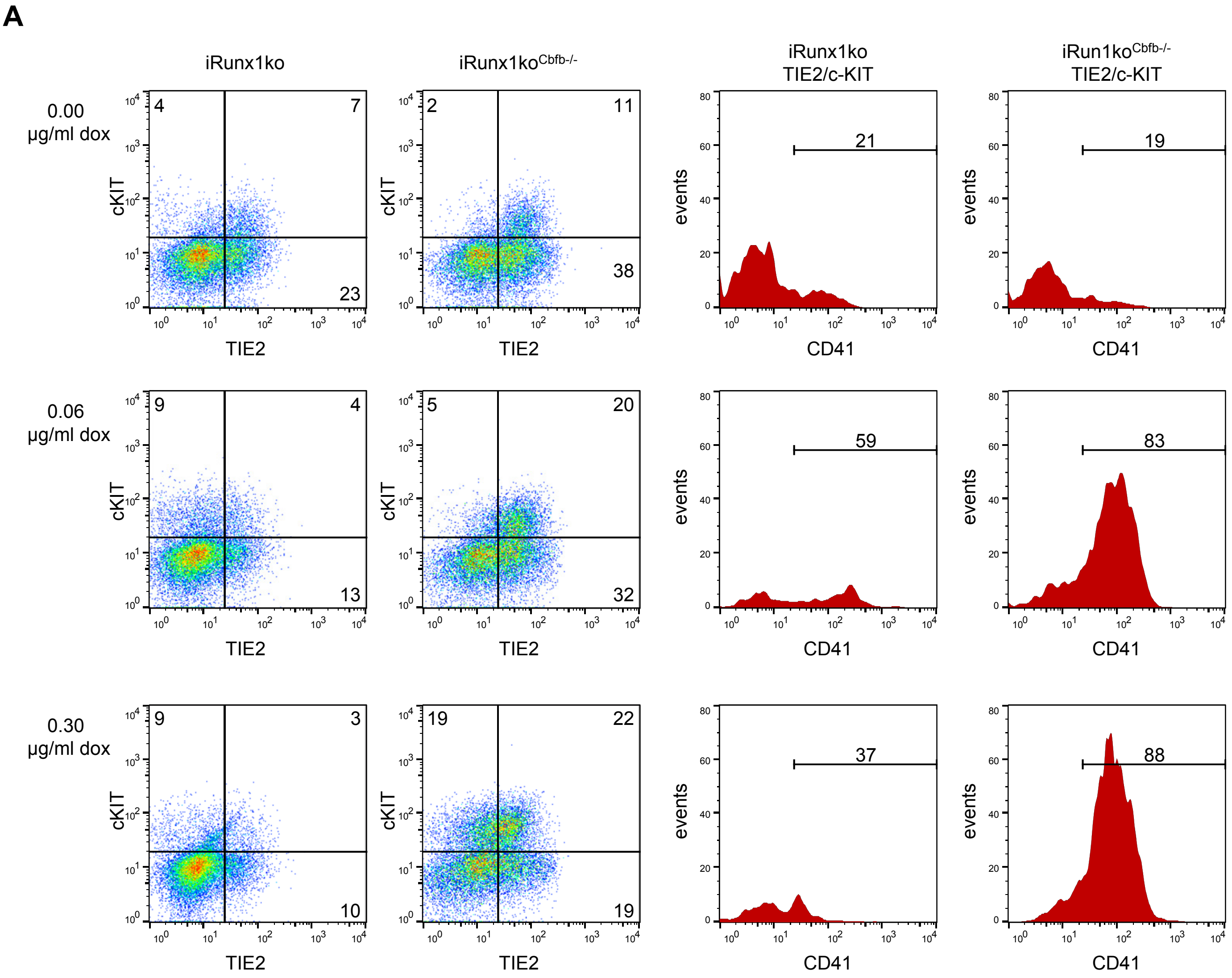

Supplemental figure 9. Persistence of HE core-like structures in doxycycline-treated iRunx1ko$^{Cbfb-/-}$ cultures. Representative flow cytometry plots of day five iRunx1ko and iRunx1ko$^{Cbfb-/-}$ EHT-cultures.

Development • Supplementary information

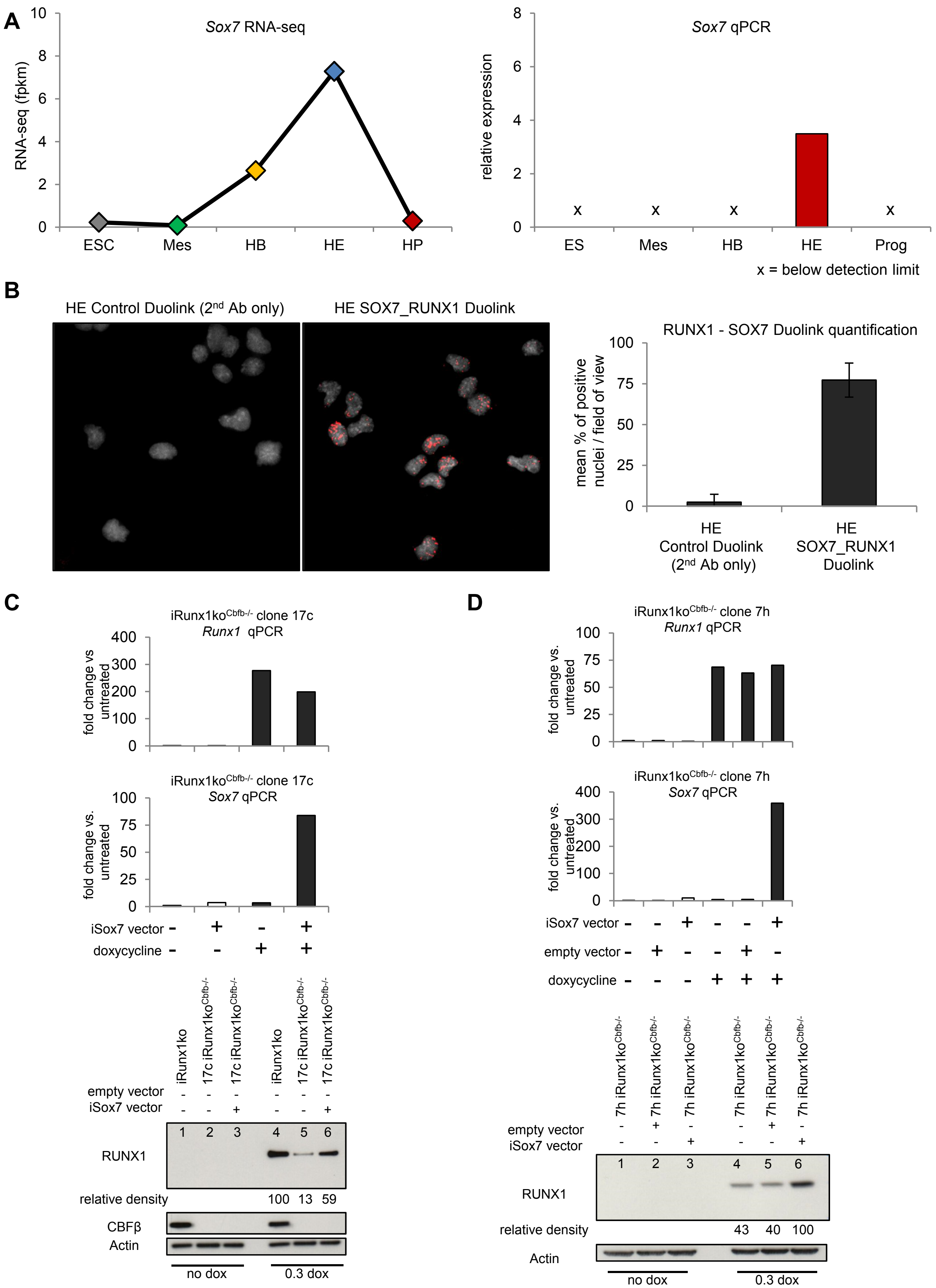
A
Sox7 RNA-seq
RNA-seq (fpkm)
ESC
Mes
HB
HE
HP
Sox7 qPCR
relative expression
ES
Mes
HB
HE
Prog
x = below detection limit
B
HE Control Duolink (2nd Ab only)
HE SOX7_RUNX1 Duolink
RUNX1 - SOX7 Duolink quantification
mean % of positive nuclei / field of view
HE Control Duolink (2nd Ab only)
HE SOX7_RUNX1 Duolink
C
iRunx1koCbfb-/- clone 17c
Runx1 qPCR
fold change vs untreated
iRunx1koCbfb-/- clone 17c
Sox7 qPCR
fold change vs. untreated
iSox7 vector
doxycycline
iRunx1ko
17c iRunx1koCbfb-/-
empty vector
iSox7 vector
RUNX1
relative density
100 13 59
CBFβ
Actin
no dox
0.3 dox
D
iRunx1koCbfb-/- clone 7h
Runx1 qPCR
fold change vs. untreated
iRunx1koCbfb-/- clone 7h
Sox7 qPCR
iSox7 vector
empty vector
doxycycline
7h iRunx1koCbfb-/-
empty vector
iSox7 vector
RUNX1
relative density
43 40 100
Actin
no dox
0.3 dox

Supplemental figure 10. CBFβ competitor SOX7 can bind RUNX1 in mESC derived HE. (A) *Sox7* expression determined by RNA-seq and qPCR across five stages of differentiation. ESC: Embryonic Stem cells, MES: Mesoderm, HB: Hemangioblast, HE: Hemogenic Endothelium, HP: Hematopoietic progenitors. Left: RNA-seq. fpkm=fragments per kilobase of transcript per million mapped reads. Right: Quantitative PCR. Mean of 5 or 6 technical replicates is shown. X=below detection limit. **(B)** Duolink assay on HE sorted SOX7-GFP cells (SOX7-GFP$^+$/TIE2$^+$/c-KIT$^+$). Left panels: Each puncta represents a single SOX7-RUNX1 interaction (40x magnification). Grey=DAPI, Red=duolink signal. Right: Percentage of SOX7-RUNX1 duolink positive nuclei in mESC derived HE. Control: mean$\pm$s.d., N=7 fields of view (137 nuclei), RUNX-SOX7: mean$\pm$s.d., N=10 fields of view (375 nuclei). **(C)** Top: qPCR in mESC for *Runx1* and *Sox7* in iRunx1ko$^{Cbfb-/-}$ clone 17c -/+ iSox7 transposon. All data is normalized against the non-induced untransfected sample. Bottom: Western blot for RUNX1, CBFβ and β-actin on mESC treated for 24 hours with or without doxycycline. iRunx1ko (lane 1, 4), iRunx1ko$^{Cbfb-/-}$ clone 17c (lane 2, 5) iRunx1ko$^{Cbfb-/-}$ clone 17c with iSox7 transposon (lane 3, 6). β-actin normalized relative density for RUNX1 is shown below the lanes. **(D)** Top: qPCR in mESC for *Runx1* and *Sox7* in iRunx1ko$^{Cbfb-/-}$ clone 7H -/+ iSox7 transposon. All data is normalized against the non-induced untransfected sample. Bottom: Western blot for RUNX1, and β-actin on mESC treated for 24 hours with or without doxycycline. iRunx1ko$^{Cbfb-/-}$ clone 7h (lane 1, 4), iRunx1ko$^{Cbfb-/-}$ clone 7h with empty transposon (lane 2, 5) and iRunx1ko$^{Cbfb-/-}$ clone 7h with iSox7 transposon (lane 3, 6). β-actin normalized relative density for RUNX1 is shown below the lanes. Inducible Sox7 lines in C & D were independently established and analyzed

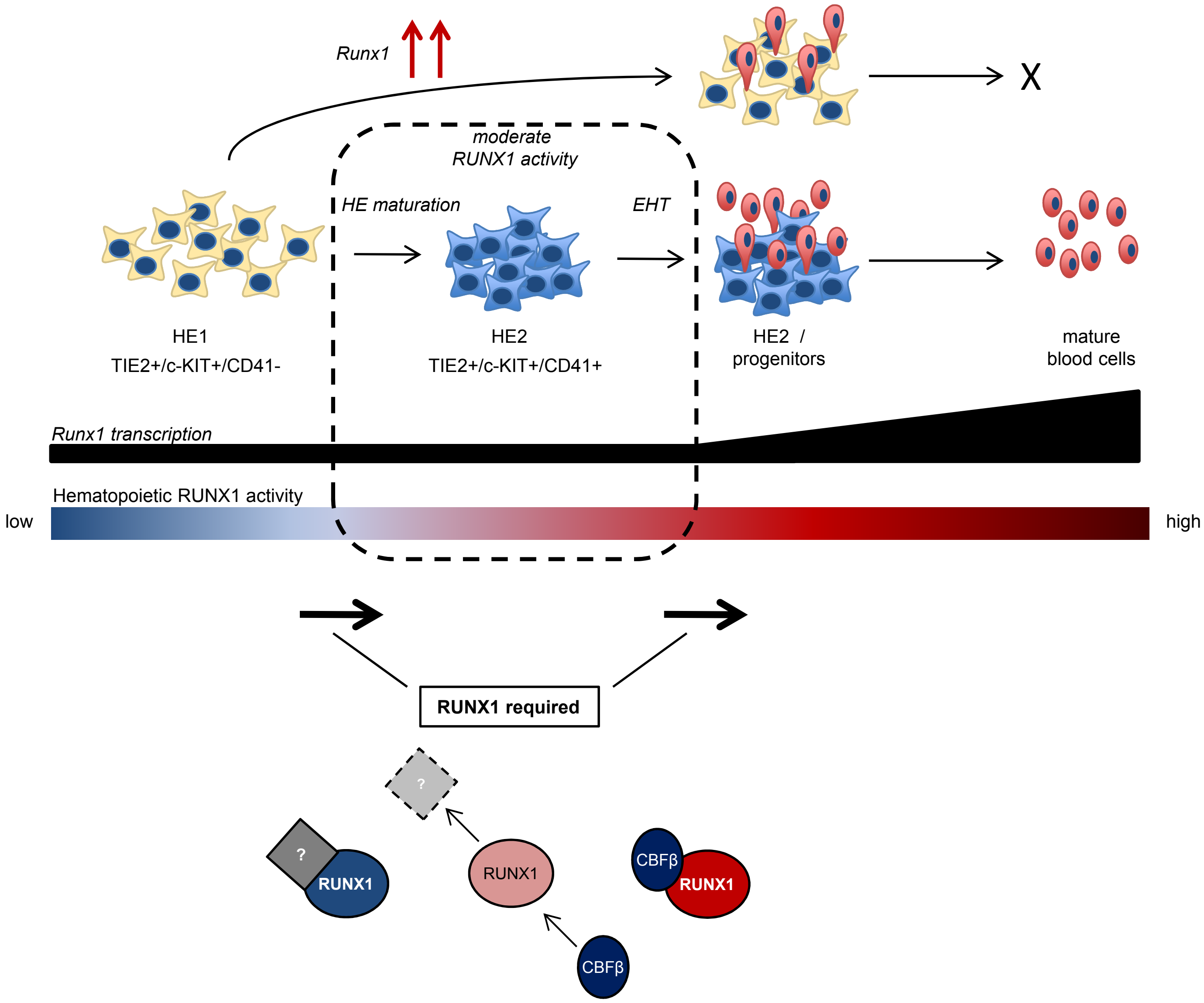

Supplemental Figure 11. RUNX1 controls the the initiation and progression of EHT. In early Hemogenic Endothelium (HE1), RUNX1 transcription and protein levels are low. An abrupt rise in HE RUNX1 levels results in an accelerated EHT but abortive blood formation. Low RUNX1 activity is required to initiate an effective EHT and mature HE1 into HE2. Once EHT is initiated RUNX1 activity is still required in order to produce mature hematopoietic cells. This may rely on an increase in *Runx1* transcription, the switch from the *Runx1b* to *Runx1c* isoform and potentially also by changing RUNX1 binding partners.

Development • Supplementary information
